# Advancing practical quantum embedding simulations *via* operator commutativity-based state preparation for complex chemical systems

**DOI:** 10.1039/d6sc03132c

**Published:** 2026-08-04

**Authors:** Dibyendu Mondal, Ashish Kumar Patra, Rahul Maitra

**Affiliations:** a Department of Chemistry, Indian Institute of Technology Bombay Powai Mumbai 400076 India rmaitra@chem.iitb.ac.in; b Qclairvoyance Quantum Labs Secunderabad TG 500094 India; c Centre for Quantum Information Computing Science & Technology, Indian Institute of Technology Bombay Powai Mumbai 400076 India

## Abstract

Determining the exponentially scaled ground state wavefunction and the associated molecular properties remains one of the central challenges in quantum chemistry. Hybrid quantum-classical algorithms implemented on quantum computers offer a promising route toward addressing this problem. However, despite several successful demonstrations on small molecular systems, accurate simulations of large and chemically realistic molecules remain difficult due to the limited capability of noisy intermediate-scale quantum (NISQ) hardware. To bypass the limitations of NISQ devices, while simultaneously retaining the accuracy of the ground state energy estimations, we propose a dynamic ansatz construction strategy based on operator commutativity and energy driven screening within the density matrix embedding theory (DMET) framework. The partitioning of the full system allows us to dynamically construct the ansatz over individual embedded subsystems, allowing each embedding problem to be solved individually to a desired accuracy. The embedding Hamiltonian is updated in a self-consistent manner with the dynamically formulated wavefunction, and their coupled optimization leads to an accurate and efficient description of the overall system. To assess the performance of this approach, we apply it to several molecular systems, including C10 (100 qubits), conformers of l-glucose (144 qubits), and the reactant, intermediate, and product structures of the methyl vinyl ketone and cyclopentadiene Diels–Alder reaction (124 qubits). These simulations require at most 20 qubits at a time and demonstrate improved accuracy and significantly reduced quantum gate requirements compared with conventional ansatze. We further investigate the impact of various fragmentation strategies and demonstrate the adaptability of our approach at each step of the DMET self-consistency cycle that leads to significantly improved accuracy for strongly correlated systems. These results highlight the emerging feasibility of addressing large-scale chemical problems of industrial relevance on near-term quantum devices.

## Abstract 2

Dynamic coupling of embedding subsystem Hamiltonians with an operator commutativity-based adaptive ansatz construction enables efficient and accurate simulation of large chemical systems on NISQ architectures.

## Graphical abstract

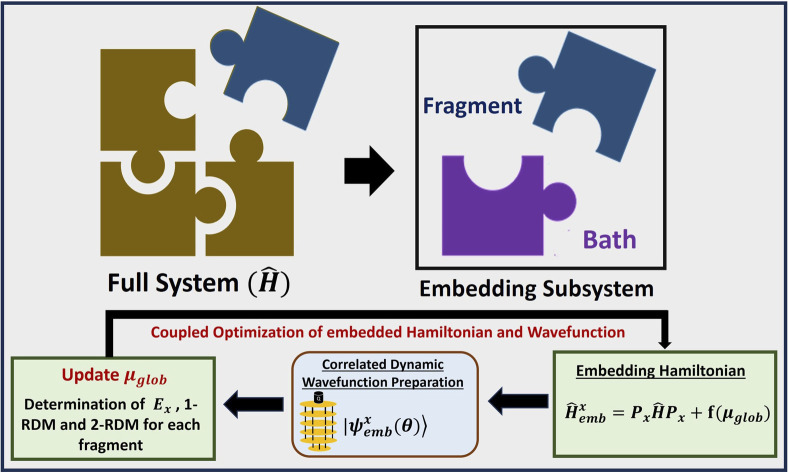

## 1. Introduction

Accurate treatment of electronic correlation remains one of the central challenges in quantum chemistry. In classical many-body electronic structure methods, an exact representation of the many-electron wavefunction requires an exponentially growing number of determinants, which becomes computationally prohibitive for classical computers beyond a certain problem size.^1,2^ Quantum computing, through the principles of quantum superposition and entanglement, provides a promising route to represent such an exponentially large Hilbert space using only a polynomial number of qubits. Nevertheless, despite this theoretical advantage, the simulation of large and realistic chemical systems is still beyond the capabilities of present day quantum hardware because of the limited number of fault-tolerant qubits and restricted gate fidelities.^3–6^ In the current noisy intermediate-scale quantum (NISQ) era, hybrid quantum-classical approaches such as the variational quantum eigensolver (VQE)^7^ have demonstrated encouraging results for small molecular systems. However, accurate simulation of realistic chemical systems would require hundreds to thousands of qubits along with significantly improved gate fidelities, which remain beyond the reach of existing quantum devices.

Within the VQE framework, strong correlation effects in small to moderately sized molecular systems can be captured by preparing a parameterized trial state or ansatz,^8,9^ (|*ψ*(*θ*)〉 = *U*(*θ*)|*ϕ*_ref_〉), starting from a suitably chosen and easy-to-prepare reference state. The parameters of the ansatz are iteratively optimized using a classical routine until the expectation value of the molecular Hamiltonian (*Ĥ*) reaches convergence.

1 *E*(*θ*) = 〈*ψ*(*θ*)|*Ĥ*|*ψ*(*θ*)〉

According to the Rayleigh–Ritz variational principle, the optimized energy obtained from this procedure provides an upper bound to the true ground state energy of the system. Consequently, both the accuracy of the computed ground state energy and the associated computational cost depend strongly on the choice of the trial ansatz. Considerable efforts have been devoted to designing compact and chemically accurate ansatze within the unitary coupled cluster (UCC) framework, which are capable of capturing strong correlation effects with manageable circuit depths.^10–19^ However, most of these approaches have primarily been demonstrated for molecular systems of limited size.

In realistic large chemical systems, the electronic structure typically involves a mixture of strong and weak correlation effects. If the strongly correlated component can be efficiently treated within the quantum computing framework, it becomes possible to capture the essential chemistry of the system in a computationally efficient manner. In this context, several classical embedding and fragmentation techniques (density matrix embedding theory (DMET),^20–22^ dynamical mean-field theory (DMFT),^23,24^ localized active space self-consistent field (LASSCF),^25,26^ fragment molecular orbital (FMO) theory,^27–29^ density functional theory (DFT) embedding,^30,31^ bootstrap embedding,^32–34^ tensor network approaches,^35,36^ and perturbative methods^37^) have been developed, where a large system is partitioned into smaller subsystems to treat correlation more effectively. Despite their success, the accuracy of these techniques often depends critically on the fragment size. As the fragment becomes larger, highly accurate classical methods for treating strong correlation such as full configuration interaction (FCI) and coupled cluster (CC) methods become computationally prohibitive. This limitation motivates the integration of efficient quantum computing algorithms to treat the strongly correlated fragments more effectively.^38^

To address these challenges, we adopt a hybrid strategy in which a large chemical system is first partitioned into smaller fragments at the mean-field level within the classical DMET framework.^20,22^ Subsequently, the localized electronic correlation within each fragment is captured by the construction of a dynamic ansatz within the UCC framework.^8,13^ This ansatz is generated through a novel operator commutativity and energy screening procedure, where higher-rank terms are tensor decomposed into effective two-body cluster operators and ‘scatterers’ to achieve a balanced description of many-body correlation effects, as introduced previously by some of the current authors.^12,13,18,39^ The choice of these scatterers is guided by their non-commutativity with the cluster operators and their energetic relevance. This methodology, termed COMmutativity Pre-screened Automated Selection of Scatterers (COMPASS),^13^ has been shown to dynamically balance accuracy with quantum efficiency *via* disentangled construction of unitaries. This structure bears a close resemblance to the contracted Schrödinger equation (CSE),^40^ whose solution provides a rapidly convergent parametrization of the wavefunction through a product of two-body exponential transformations. Its anti-Hermitian form (ACSE)^41,42^ provides a theoretically exact unitary representation of the wavefunction that is naturally suited for quantum computation. The novel contracted quantum eigensolver (CQE)^43,44^ further exploits this framework by solving the ACSE through minimization of the residual of the Schrödinger equation projected onto the two-electron space. This framework alleviates the need for deep quantum circuits and extensive classical optimization in obtaining ground-state energies. The practical applicability of CQE on noisy quantum hardware was further improved through *N*-representability constrained 2-RDM projection^45^ and a shadow tomography-based ansatz^46^ that reduces measurement cost while preserving exactness. In a similar spirit, in the COMPASS protocol, the circuit depth challenge is addressed with a minimally parametrized disconnected operator block formation that requires multiple shallow circuit optimizations, as explained in the next section.

In the DMET framework, which provides a practical platform for studying large chemical systems by decomposing them into manageable fragments that are compatible with the limitations of current NISQ hardware, the adaptation of COMPASS is far from trivial. This is primarily due to the non-trivial coupling between the embedding Hamiltonians and the dynamically constructed ansatz for each subsystem. This complexity arises because within DMET, the consistency between the high-level correlated wavefunction and the low-level mean-field description is enforced through the introduction of a global chemical potential. This chemical potential is incorporated into each embedded Hamiltonian and iteratively optimized such that the total particle number obtained from all fragmented subsystem's high-level wavefunction matches that of the mean-field wavefunction of the full system. Thus, during this self-consistent optimization cycle, the dynamic high-level correlated wavefunction for each fragment must be reconstructed at every step with the updated chemical potential to ensure a consistent and balanced description across all subsystems. Thus, in our proposed scheme, the dynamic nature is not only at the level of ansatz preparation for embedding subsystems, but also at the level of the embedding Hamiltonians, which get modified at each step, requiring an alternative optimization cycle for ansatze and the Hamiltonians. This formalism is abbreviated as DMET-COMPASS.

The rest of the manuscript is organized as follows: in Section 2.1, we begin with an overview of the DMET framework, followed by the construction of high-level wavefunctions on a quantum computer in Section 2.2. The efficient dynamic ansatz construction strategy is discussed in Section 2.3, followed by a detailed description of the hybrid quantum-classical embedding framework in Section 3. To validate the proposed approach, we present numerical results in Section 4 for a range of molecular systems, including the circular C_10_ system, multiple conformers of l-glucose, and the reaction energy profile of the (4 + 2) cycloaddition between cyclopentadiene and methyl vinyl ketone. These systems require 100, 144, and 124 qubits, respectively, for full system simulations on a quantum computer within the STO-3G basis. By employing an atom-wise partitioning strategy within the DMET framework, the effective problem size is reduced to at most 20 qubits. In Section 4.5, we systematically analyze the effects of various fragmentation sizes with the strongly correlated linear H_12_ system and demonstrate how the size of the embedding problem critically impacts the performance of DMET-COMPASS. Finally, in Section 4.6, we demonstrate the adaptability of the ansatz during the self-consistent iterative evolution of the embedding Hamiltonian and show that the coupled optimization of the high-level wavefunction of the embedded subsystems, along with the recursive update of the embedding Hamiltonian, leads to improved description of the strongly correlated molecular systems.

## 2. Theory

### 2.1. Density matrix embedding framework

In the quantum computing paradigm, each spin–orbital is usually mapped directly onto a qubit. Consequently, simulating a large chemical system demands an enormous number of fault-tolerant qubits, far exceeding the capabilities of present-day quantum hardware. To make realistic quantum chemical simulations feasible, it is therefore essential to minimize qubit requirements.

One promising strategy is the use of quantum embedding methods, among which DMET^20,21^ is particularly powerful. DMET reformulates the original quantum problem into a reduced system consisting of a fragment, its entangled bath, and the surrounding pure environment. The central idea is to compress the Hilbert space by applying a Schmidt decomposition to the wavefunction. Concretely, the Hilbert space is partitioned into two orthogonal subspaces: the fragment A with dimension *L*_A_ and environment B with dimension *L*_B_, (*L*_B_ > *L*_A_). The full quantum state in the product basis |*A*_*i*_〉|*B*_*j*_〉, which spans a dimension of (*L*_A_ × *L*_B_), can then be expressed as:2
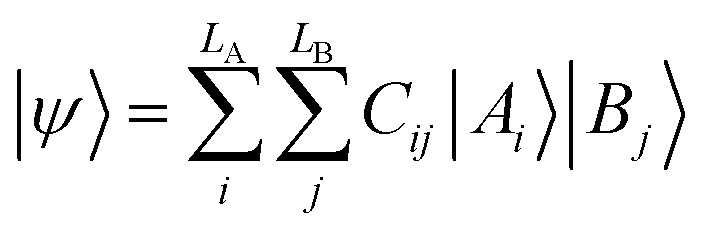
where *C*_*ij*_ is the coefficient tensor. However, the effective size of the problem can be significantly reduced by explicitly accounting for the entanglement between the fragment and the environment. In particular, the quantum state |*ψ*〉 can be decomposed into a rotated basis as:3
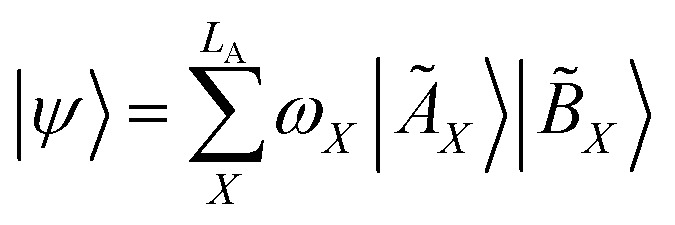
which corresponds to the Schmidt decomposition of bipartite states. This decomposition naturally partitions the environment into two distinct parts: a set of at most *L*_A_ bath states entangled with the fragment and the remaining states that are completely disentangled. On this basis, the embedding Hamiltonian can be constructed by projecting the full Hamiltonian (*Ĥ*) into the reduced subspace defined by the fragment and its associated bath. This is achieved through the relation *Ĥ*_emb_ = *P̂ĤP̂* with the projector *P̂* defined as:4
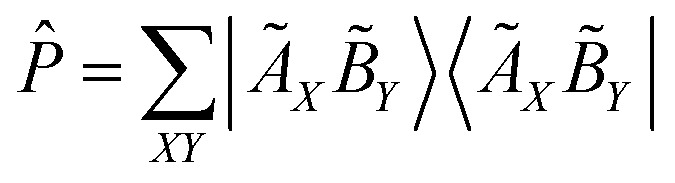
which is the projection onto an at most 2*L*_A_ dimensional subspace, significantly smaller than the full space. Although the constructed subspace is formally exact and demonstrates that the full system can, in principle, be represented by a fragment coupled to its entangled bath, it remains impractical in practice. This is due to the fact that it relies on prior knowledge of the exact ground state wavefunction and its corresponding Schmidt decomposition, which makes it somewhat impractical. In practice, DMET employs a mean-field approximation to the ground state wavefunction of the full system and generates the fragment and bath orbitals, which together form a partitioned subsystem (*ξ*). Within each embedded fragment-bath space, the mean-field state (|*ϕ*^*ξ*^_ref_〉) serves as a reference, and electron correlation may be incorporated through high-level correlation calculation using a suitable parametrized quantum circuit (|*ψ*^*ξ*^(*θ*_opt_)〉).

Since each subsystem is treated independently, the redistribution of electrons between fragment and bath orbitals during the DMET procedure can lead to an inconsistency in the electron count. As a result, the total number of electrons obtained by summing over all fragments may deviate from that of the full system. To enforce particle number consistency, a global chemical potential (*µ*_gl_) is introduced, which modifies the embedded Hamiltonian (*Ĥ*^*ξ*^_emb_) as:5
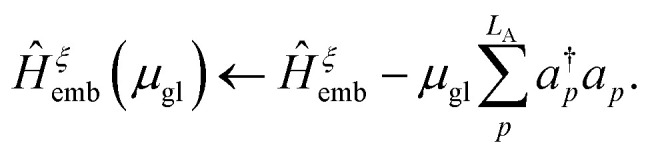


After obtaining the correlated high-level wavefunction for each embedded Hamiltonian (*Ĥ*^*ξ*^_emb_(*µ*_gl_)), the one- 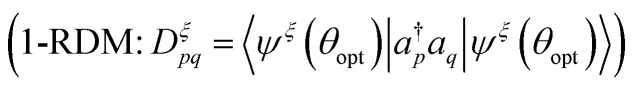 and two-particle reduced density matrices 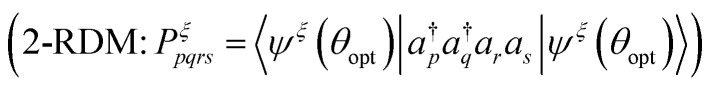 are calculated with the high level wavefunction. These reduced density matrices are then used to evaluate the energy of each embedded subsystem:6
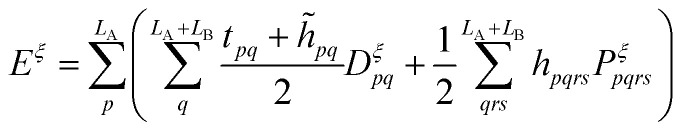
where 
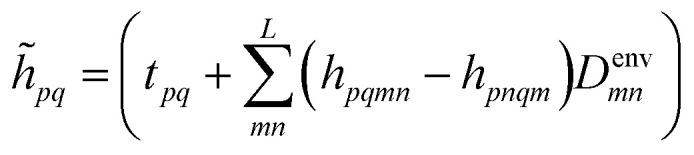
 represents the rotated one-electron integral, which also considers the effect of the unentangled electrons. Finally, the total energy of the entire system is calculated by summing up all the fragment energies.7
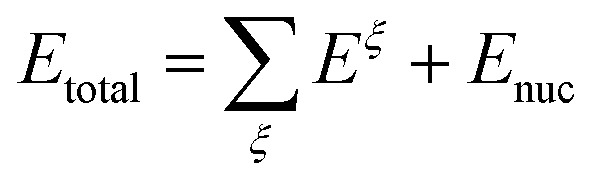
Here *E*_nuc_ is the nuclear repulsion energy of the entire system. In practice, this procedure is carried out self-consistently. Starting from an initial guess for the global chemical potential, the high-level wavefunction and corresponding electron number are computed for each subsystem. The chemical potential is then iteratively updated until the deviation between the total electron count (summed over all fragments) and that of the full system falls below a predefined threshold. Importantly, at each iteration, the embedded Hamiltonians are updated and the high-level wavefunctions are recomputed to ensure overall consistency. It is important to note that in the DMET framework, the full system Hamiltonian is partitioned into multiple embedded Hamiltonians. As a consequence, the sum of the embedded energies is not necessarily guaranteed to remain variational with respect to the exact ground state energy of the full system. However, for each embedded subsystem, if the correlated wavefunction is obtained using the VQE, the energy of an individual embedded subsystem remains variationally upper bound by the corresponding ground state energy of its embedded Hamiltonian, but not by the total energy.

### 2.2. Correlated high-level wavefunction generation on a quantum computer

The correlated high-level wavefunctions for each embedded subsystem can, in principle, be obtained using accurate classical electronic structure methods. However, the overall accuracy of energy strongly depends on the size of the fragment: as the embedded subsystem becomes larger, the computational cost of reliable classical approaches grows rapidly, often rendering them impractical. In such cases, quantum computing algorithms provide a promising alternative, offering a more scalable route to accurately capture correlation effects in large embedded spaces. As discussed earlier, given the limitations of current quantum hardware, hybrid quantum-classical approaches such as VQE are more suitable for obtaining correlated wavefunctions. The accuracy and computational efficiency of VQE are largely determined by the choice of the trial ansatz. Within the UCC framework, the trial ansatz for each embedded subsystem is constructed by applying anti-Hermitian excitation operators on top of the mean-field reference state of the embedded subsystem.

8 |*ψ*^*ξ*^(*θ*)〉 = e^*τξ*(*θ*)^|*ϕ*^*ξ*^_ref_〉

Here *τ*^*ξ*^(*θ*) = *T*^*ξ*^(*θ*) − *T*^*ξ*†^(*θ*) is the anti-Hermitian cluster operator and |*ϕ*^*ξ*^_ref_〉 is the mean field state of embedded system *ξ*. The general practice is to truncate the cluster operators at double excitations, and thus the UCC ansatz with single and double excitation operators provide a balance between accuracy and resource efficiency. However, in strongly correlated regions, higher-order excitation effects, which require a large number of quantum resources, are very important to capture the correlation effects effectively. An alternative strategy is required to capture such important phenomena.

### 2.3. Dynamic ansatz construction for the high-level wavefunction generation

Direct inclusion of higher-order excitation operators demands high quantum resources, although those effects are supremely important to generate an accurate high-level wavefunction for each embedded system. To bypass this problem, we have adopted a dynamic ansatz construction strategy where, in addition to single and double excitation operators, we have included a class of two-body generalized operators to capture the higher order correlation effects implicitly. Therefore, through our strategy, one can generate important higher order effects (localized within each embedded subsystem) through lower-body parametrization without incurring additional quantum resources.

#### 2.3.1. A class of generalized operators: scattering operators

In the case of generalized operators, excitation can occur from occupied to occupied or from unoccupied to unoccupied orbitals with respect to the mean field reference. The class of generalized operators that we have used has a specific structure. These are two-body operators with an effective excitation rank of one. In detail, there is an excitation from occupied to unoccupied orbitals of the mean field reference and an occupied-to-occupied or unoccupied-to-unoccupied scattering. Essentially, there are hole/particle destruction operators. Based on the type of destruction operator, they are classified as *S*^*ξ*^_h_ or *S*^*ξ*^_p_.9
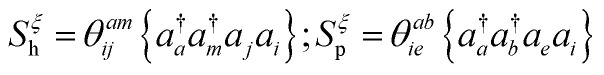
Here *i*, *j*, …*m* and *a*, *b*, …*e* denote the occupied and unoccupied orbital indices with respect to the mean field reference (|*ϕ*^*ξ*^_ref_〉) of embedded subsystem *ξ*. The introduction of scatterers essentially enables us to write every term of three or higher rank excitation operators through tensor decomposition of two-body terms: ((*S*^*ξ*^)^*am*^_*ij*_(*T*^*ξ*^)^*bc*^_*mk*_ → (*T*^*ξ*^)^*abc*^_*ijk*_ ← (*S*^*ξ*^)^*ab*^_*ie*_(*T*^*ξ*^)^*ec*^_*jk*_), which are localized in *ξ*. The orbitals (*m* ∈ *ξ* in *S*^*ξ*^_h_ and *e* ∈ *ξ* in *S*^*ξ*^_p_) through which they contract with excitation operators are known as the contractible set of orbitals (CSOs). In the present work, the CSOs are restricted to the HOMO and LUMO of embedding subsystems for all applications. Since the HOMO–LUMO gap generally corresponds to the smallest energy separation in the system, excitation operators involving these orbitals are expected to possess relatively large amplitudes from perturbative considerations. Consequently, such operators are more likely to contribute to dominant pathways for generating higher-rank correlation effects through scattering. It is very important to note that the direct action of scatterers on the mean field reference is nilpotent *S*^*ξ*^|*ϕ*^*ξ*^_ref_〉 = 0. Thus, the excitation operators and scatterers are chosen to be non-commutative partners of each other such that their combined effective action over the reference is nontrivial. The anti-Hermitian counterpart of higher order effects is generated through this non-commutativity:

10 [*σ*^*ξ*^, *τ*^*ξ*^_2_] → *τ*^*ξ*^_3_; [*σ*^*ξ*^, [*σ*^*ξ*^, *τ*^*ξ*^_2_]] → *τ*^*ξ*^_4_…

Here *σ*^*ξ*^ = *S*^*ξ*^ − (*S*^*ξ*^)^†^ and *τ*^*ξ*^ represent the anti-Hermitian scatterers and excitation operators of embedded subsystem *ξ*, respectively. The disentangled product of exp(*σ*^*ξ*^) and exp(*τ*^*ξ*^), as they appear in an interwoven manner in our ansatz (see the next section), introduces the exponential of an effective higher rank excitation operator through the commutator. As the rank of commutators grows, the corresponding excitation ranks increase accordingly. While, in principle, higher-order effects can be constructed solely from rank-two operators, incorporating the full set of one and two-body excitation operators along with two-body scattering terms demands substantial quantum resources, exceeding the limits of current quantum hardware. Moreover, the realization of higher-order contributions is highly sensitive to the ordering of the chosen excitation and scattering operators. Therefore, an efficient and systematic strategy is essential to selectively screen the relevant cluster and scattering operators, as well as to determine their optimal ordering. We present such a strategy in the next subsection.

#### 2.3.2. COMPASS – a dynamic ansatz tailoring strategy for embedded subsystems

The selection of dominant excitation operators and scatterers, as well as their ordering in the trial ansatz, play a very crucial role in determining an accurate high-level wavefunction for each embedded subsystem. More importantly, the ordering of the operators and/or their structural orientation may vary for each embedded subsystem, requiring local optimization and construction of the ansatz for each subsystem. To address this, in the first step of our ansatz construction scheme, we systematically prune the relevant two-body excitation operators through a parallel single-parameter optimization procedure. Starting from the mean-field reference state (|*ϕ*^*ξ*^_ref_〉) of the embedded system *ξ*, we construct a set of trial circuits by applying each two-body excitation operator individually. Each of these single parameter circuits is then independently optimized to obtain the corresponding minimum energy, enabling the identification of the most significant operators.

11 *E*^*ξ*^_*I*_ = 〈*ϕ*^*ξ*^_ref_|e^−*τ*_*I*_^*ξ*^(*θ*_*I*_)^*Ĥ*^*ξ*^_emb_(*µ*_gl_)e^*τ*_*I*_^*ξ*^(*θ*_*I*_)^|*ϕ*^*ξ*^_ref_〉

Here *I* represents the combined hole–particle indices of two-body excitation operators of embedded subsystem *ξ*. Only those two-body excitation operators are included for which the energy difference Δ*E*^*ξ*^_*I*_ = (*E*^*ξ*^_ref_ − *E*^*ξ*^_*I*_) from the mean field reference state is greater than a predefined threshold Δ*E*^*ξ*^_*I*_ > *ε*_1_. The selected two-body excitation operators are placed into several operator blocks (*α*, *β*, *γ*… ∈ *ξ*) in descending order of their energy contribution to the reference state. This implies that the energetically most dominating two-body operator is allowed to act on the reference state directly. This means that, among the selected two-body excitation operators, if Δ*E*^*ξ*^_*I*_… > Δ*E*^*ξ*^_*J*_… > Δ*E*^*ξ*^_*K*_…, the corresponding operators are placed in operator blocks *α*, *β* and *γ*, respectively, where … > *γ*… > *β*… > *α*… > 1. This sequence will be referred to as the ‘ordering’ of the operator blocks, which indicates their relative positions with respect to the reference state in the unitary circuit and it remains fixed throughout. After this step, the ansatz structure is as follows:

12 |*ψ*^*ξ*^(*θ*)〉 = …*U*^*ξ*^_*γ*_…*U*^*ξ*^_*β*_…*U*^*ξ*^_*α*_…|*ϕ*^*ξ*^_ref_〉

where *U*^*ξ*^_*α*_ = [e^*τ*_*I*_^*ξ*^(*θ*_*I*_)^]_*α*_. In the subsequent step, each operator block is further enriched by incorporating suitable scattering operators. For every selected two-body excitation operator within a given block, we sequentially examine the non-commutativity of potential scatterers based on the presence of common CSOs with that excitation operator. A scatterer sharing common CSOs effectively generates higher-order excitation effects. We then assess the significance of these generated triple-excitation contributions by evaluating their impact on the energy. This is achieved through the optimization of a corresponding two-parameter quantum circuit.13
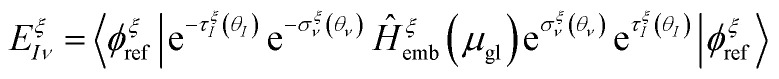
Here *ν* represents the combined hole–particle indices of scatterers of embedded subsystem *ξ*. Now only those scatterers are selected and added to the corresponding operator block for which the energy difference Δ*E*^*ξ*^_*Iν*_ = (*E*^*ξ*^_*I*_ − *E*^*ξ*^_*Iν*_) is greater than predefined threshold (Δ*E*^*ξ*^_*Iν*_ > *ε*_2_).

In order to explain the grouping of operators and assigning their relative ordering, let us consider a pedagogic example. Let us consider a case where the scattering operator *σ*^*ξ*^_*ν*_ satisfies (non-)commutativity with *τ*^*ξ*^_*I*_ while simultaneously being selected by the energy criterion (eqn (13)). This will be paired with *τ*^*ξ*^_*I*_ in its originally assigned operator block *U*_*α*_. Similarly, let us assume that both the scattering operators *σ*^*ξ*^_*ν*_ and *σ*^*ξ*^_*λ*_ satisfy the energy criterion and are non-commuting with the cluster operator *τ*^*ξ*^_*K*,_ which was previously selected in the block *U*_*γ*_. On the other hand, suppose no scattering operator fulfills at least one of the criteria to be paired with the operator *τ*^*ξ*^_*J*_ in the block *U*_*β*_. Consequently, only the operator blocks *U*_*α*_ and *U*_*γ*_ will be expanded as:*U*_*α*_ = [e^*σ*_*ν*_^*ξ*^(*θ*_*ν*_)^e^*τ*_*I*_^*ξ*^(*θ*_*I*_)^]_*α*_; *U*_*γ*_ = [e^*σ*_*λ*_^*ξ*^(*θ*_*λ*_)^e^*σ*_*ν*_^*ξ*^(*θ*_*ν*_)^e^*τ*_*K*_^*ξ*^(*θ*_*K*_)^]_*γ*_while *U*_*β*_ = [e^*τ*_*J*_^*ξ*^(*θ*_*J*_)^]_*β*_. As previously mentioned, it is important to note that the ordering of the operator blocks selected after eqn (12) remains unchanged in this step; only the dominant scatterers are incorporated into their respective blocks. Subsequently, all single excitation operators are appended at the final stage. Since the quantum resource requirements associated with single excitation operators are relatively minimal, no additional screening criteria are imposed to limit their number. The resulting construction yields the final form of the trial high-level wavefunction for each embedded subsystem *ξ*.14
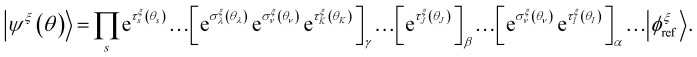
Here single excitation operators are represented by ‘*s*’. Once the trial ansatz is fully constructed, by optimizing the variational parameters of the same, eqn (14), one obtains the correlated high-level wavefunction along with the corresponding minimum energy for the embedded subsystem *ξ*. Repeating this procedure independently for each fragment enables the evaluation of the correlated energies and wavefunctions across all subsystems. Here, one may note that the length and structure of the ansatz vary for different embedded subsystems. Having discussed the building blocks of our theoretical formulation, in the next section, we present a stepwise methodology that leads to the dynamic construction of the high-level wavefunction of the embedded problem and the corresponding energy optimization.

At this juncture, it is important to establish the connection of COMPASS-VQE and its factorized ansatz structure with other allied theories. The factorized form, as appears in COMPASS, is crucial for the exactness of the wavefunction and is reminiscent of the contracted Schrödinger equation (CSE).^40^ The CSE and its anti-Hermitian variant (ACSE)^41,42^ enable direct evaluation of energies and two-electron reduced density matrices, offering greater efficiency and expressibility. A notable development in this direction is the CQE,^43,44^ which solves the ACSE by minimizing the residual of the *N*-electron Schrödinger equation projected onto the two-electron space. Unlike COMPASS, which grows the ansatz through concatenation of operator blocks, CQE is structurally closer to adaptive derivative-assembled pseudo-Trotterization VQE (ADAPT-VQE).^11^ However, they carry one important difference: while CQE iteratively constructs and updates a compact wavefunction representation from the ACSE residual, ADAPT-VQE employs a gradient-based operator selection strategy. Since this residual also corresponds to the energy gradient with respect to two-body unitary transformations, CQE provides a natural and theoretically exact framework for wavefunction optimization. However, as previously mentioned, unlike both CQE and ADAPT-VQE, COMPASS constructs the full ansatz through operator commutativity and a series of shallow-circuit VQE optimizations.

## 3. Methodology

(a) Low-level description of the full system: the mean-field wavefunction of the entire system is obtained using the Restricted Hartree–Fock (RHF) approach (appropriate for closed-shell systems, which are considered here). This calculation provides the molecular orbital coefficient matrix along with and the total electron count, which serve as the fundamental inputs for subsequent partitioning of the system.

(b) Fragment orbital construction: the full system is partitioned into multiple user-defined, atom-centered fragments (A_y_). For each fragment, localized and orthonormalized molecular orbitals are generated using localization techniques (here we use the meta-Lowdin localization scheme), forming the corresponding fragment orbital basis.

(c) Construction of entangled bath orbitals: for every fragment, bath orbitals (B_y_) are identified from the remaining environmental orbitals that exhibit entanglement with the fragment space. This is determined based on eigenvalues lying between 0 and 1 obtained *via* diagonalizing the environment subblock of one-body reduced density matrix. Orbitals with eigenvalues close to 1 are treated as fully occupied, while those with eigenvalues below 10^−13^ are classified as virtual and excluded from the embedding space. The combination of fragment and bath orbitals (A_y_ + B_y_) defines the embedded subsystem *ξ*.

(d) DMET macro cycle:

• Embedded Hamiltonian formation: for each subsystem *ξ*, an effective Hamiltonian is constructed by projecting the full system Hamiltonian onto the fragment plus bath orbital space. A global chemical potential (*µ*_gl_) term is incorporated and adjusted to ensure particle number conservation, thereby enforcing self-consistency across all embedded subsystems (eqn (5)).

• Dynamical design of the high-level wavefunction using the embedded Hamiltonian: starting from the mean-field reference state and the embedded Hamiltonian for each subsystem *ξ*, a correlated high-level wavefunction is constructed by systematically building a compact and dynamic parametrized ansatz through the following procedure:

i. One-parameter local VQE micro-cycles: for each subsystem *ξ*, the dominant two-body excitation operators are identified through single-parameter VQE optimization cycles. Based on their individual energy contributions relative to the mean-field reference, the selected operators are organized into different operator blocks (eqn (11)).

ii. Commutativity-based operator selection for higher-order effects: for the selected two-body excitation operators within individual operator blocks, suitable scatterers are chosen by analyzing commutativity relations or commonality of the corresponding CSOs. The restriction of CSOs requires us to identify the active orbitals for each embedding problem. This enables the effective incorporation of higher-order (*e.g.*, triple) excitation contributions through nested commutators.

iii. Two-parameter local VQE micro-cycles: the most significant triple excitation effects are further screened *via* optimizing two-parameter VQE circuits, each composed of one two-body excitation operator and one non-commuting scatterer. This step ensures inclusion of essential correlation effects while maintaining reduced circuit complexity (eqn (13)).

iv. Global VQE optimization cycle: the final ansatz for each subsystem, comprising the selected two-body operators, associated scatterers, and all single excitation operators, is optimized variationally. The optimization is initialized using parameter values obtained from the preceding local screening cycles to enhance convergence efficiency.

15 *E*^*ξ*^(*θ*) = 〈*ψ*^*ξ*^(*θ*)|*Ĥ*^*ξ*^_emb_(*µ*_gl_)|*ψ*^*ξ*^(*θ*)〉; *θ* ∈ *ξ*

(e) Energy and electron number evaluation for each embedded subsystem: from the correlated high-level wavefunction of each embedded subsystem, the one- and two-particle reduced density matrices (1-RDM and 2-RDM) are evaluated. These quantities are then used to compute the energy of each subsystem. The electron population within a given fragment is obtained as 
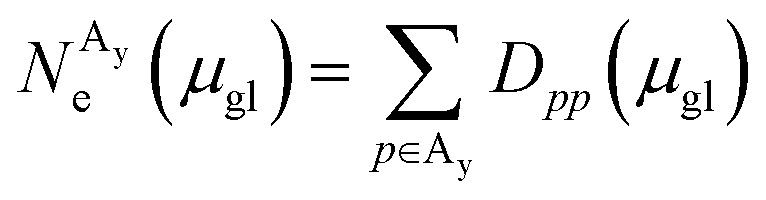
, where *D*_*pp*_(*µ*_gl_) represents the diagonal elements of the 1-RDM. The total electron number across all fragments is subsequently determined by summing the individual fragment contributions:16
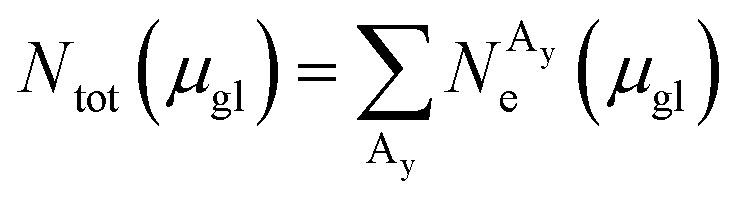


(f) Self-consistency check: the consistency of the electron number is assessed by evaluating the deviation Δ*N*_e_ = *N*_tot_(*µ*_gl_) − *N*_act_, where *N*_act_ denotes the total number of electrons in the full system. If Δ*N*_e_ falls below a predefined tolerance, the procedure is terminated and the total energy of the system is computed. Otherwise, the global chemical potential *µ*_gl_ is updated, the embedded Hamiltonians are reconstructed accordingly, and the subsequent steps (starting from step d) are repeated until convergence is achieved.

Below, we discuss a few pilot applications to realistic chemical systems that convincingly prove the superiority of the dynamic high-level wavefunction construction strategy.

## 4. Results and discussion

### 4.1. General considerations

In all the simulations, we have used Tangelo,^47^ which imports one- and two-electron integrals from PyScf^48^ and creates fragment and bath orbitals. For the calculation of the high-level wavefunction, we have integrated Qiskit-nature^49^ into the Tangelo framework. In all the test cases, we have used the STO-3G basis^50^ set and employed Jordan–Wigner encoding for fermionic to qubit mapping.^51^ In all the test cases, we have employed energy thresholds of 10^−5^ and 10^−7^ for operator selection in the one-parameter and two-parameter local VQE micro-cycles, respectively, for each embedded subsystem. To reduce the number of parameters, we have considered two specific sets of scattering operator pools. In the first pool, we include only those two-body scattering operators in which the transition occurs either from the same spatial orbitals (for *S*_h_ type scatterers) or into the same spatial orbitals (for *S*_p_ type scatterers) of the mean-field reference of the embedded subsystem. This set is referred to as the partial-pairing scattering operator pool. In the second pool, we consider only those scattering operators for which the excitation and scattering vertices belong to different spin sectors with respect to the mean-field reference of the embedded subsystem. This set is termed the opposite-spin scattering operator pool. For both pools, the corresponding CSOs are restricted to the HOMO and LUMO of the embedded subsystem's mean-field reference. In the VQE optimization, we have used the SLSQP optimizer (as implemented in the SCIPY library).^52^ Furthermore, for the convergence of the DMET cycle, we impose a threshold of Δ*N*_e_ = 10^−5^ in all test simulations. In the DMET cycle, we have used the Newton-secant root finding method to update the global chemical potential.^53^ In the case of ADAPT-VQE^11^ simulations, we have employed a gradient threshold of 10^−4^. This implies that, during the construction of the high-level wavefunction for each embedded subsystem, operators corresponding to the largest gradient are iteratively added to the ansatz until the maximum gradient falls below the specified threshold. For these ADAPT-VQE simulations, we have used a single- and double-excitation operator pool.

### 4.2. Potential energy profile of cyclo[10]carbon (C_10_)

**Fig. 1:**
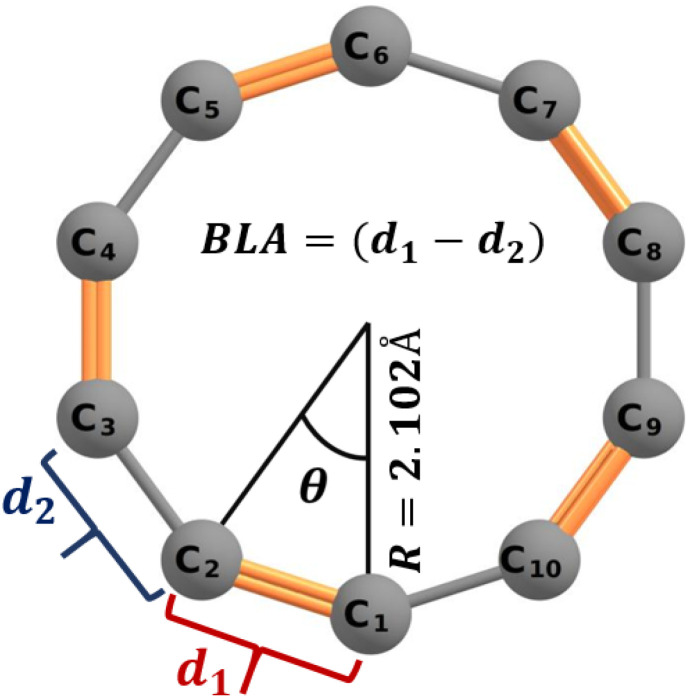
A schematic structure of planar C_10_ is presented. The angle between two nearest carbon atoms is defined by *θ*. The two types of C–C bond lengths are presented *via d*_1_ and *d*_2_, and their difference (*d*_1_–*d*_2_) defines bond length alternation (BLA).

Following the discovery of carbon allotropes such as fullerenes, carbon nanotubes, and graphene, another intriguing class of all-carbon systems, cyclo[*n*]carbons (*C*_*n*_), has attracted significant attention due to its unique structural and electronic properties. In this work, we investigate various geometries of cyclic C_10_ by systematically varying the bond angle (*θ*) between adjacent carbon atoms. All carbon atoms are constrained to lie in a single plane. The structure is characterized by two distinct C–C bond lengths (*d*_1_ and *d*_2_), and their difference defines the bond length alternation (BLA: (*d*_1_ − *d*_2_)). A structural demonstration of the system is presented in Fig. 1.

**Fig. 2:**
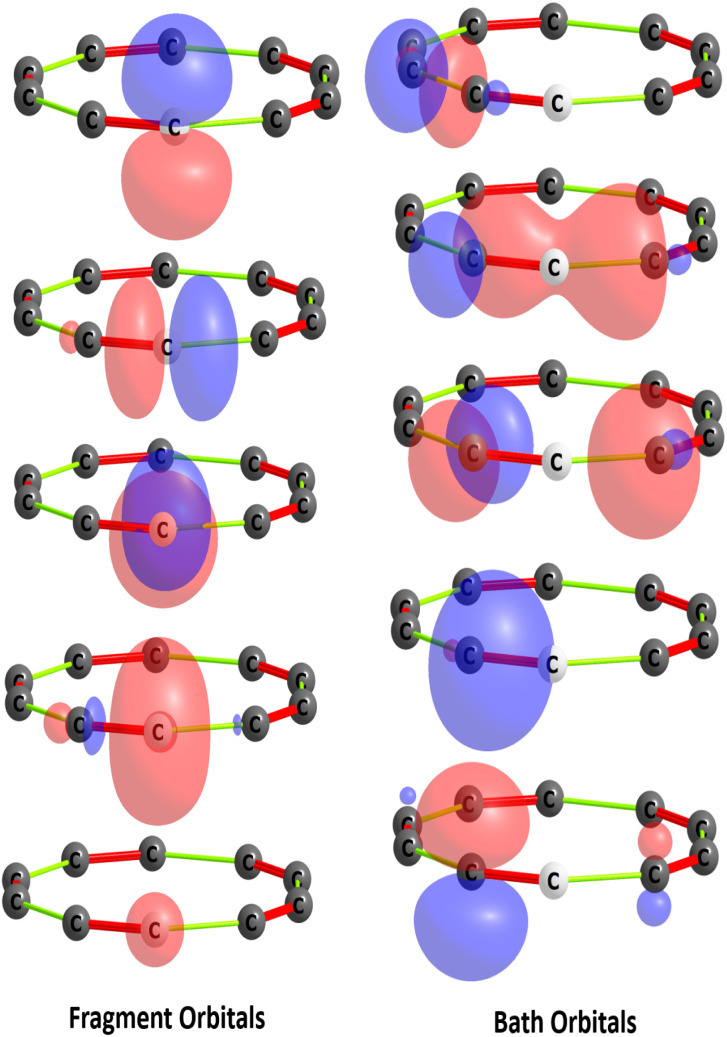
The fragment and bath orbitals are presented for C_10_ obtained by partitioning the full system into 10 equivalent C atoms. The orbitals correspond to the carbon atom that is colored white.

At *θ* = 36°, the BLA becomes zero, leading to a cumulenic structure with *D*_10h_ symmetry. For all other values of *θ*, the system adopts a polyynic structure with *D*_5h_ symmetry and exhibits a non-zero BLA. In our study, the radius (*R*) of the cyclic C_10_ ring is fixed at 2.102 Å. The VQE simulation of the full C_10_ system in the STO-3G basis requires 100 qubits.

To analyze the system within the embedding framework, we partition the molecule into 10 fragments using an atom-centered decomposition. The fragment molecular orbitals and the corresponding entangled bath orbitals for a carbon fragment of C_10_ are shown in Fig. 2. For each fragment, an embedded Hamiltonian is constructed, and the corresponding energy is evaluated by preparing high-level correlated wavefunctions using the COMPASS-VQE approach, as well as standard UCCSD and UCCSDT ansatze, within the iterative DMET self-consistency cycle. In this study, the high-level wavefunction for each fragment is calculated in a (6,6) active space, keeping the two core occupied and two high-lying unoccupied orbitals of the embedded subsystem frozen. Thus, the simulation of the high-level wavefunction on a quantum computer for each embedded subsystem requires 12 qubits. The obtained results are benchmarked against DMET-FCI calculations within that active space, where the high-level wavefunction for each embedded subsystem is determined using the exact FCI method at every step of the global chemical potential optimization. For the DMET-COMPASS simulation, we have used the partial-pairing scattering operator pool (see subsection 4.1).

**Fig. 3:**
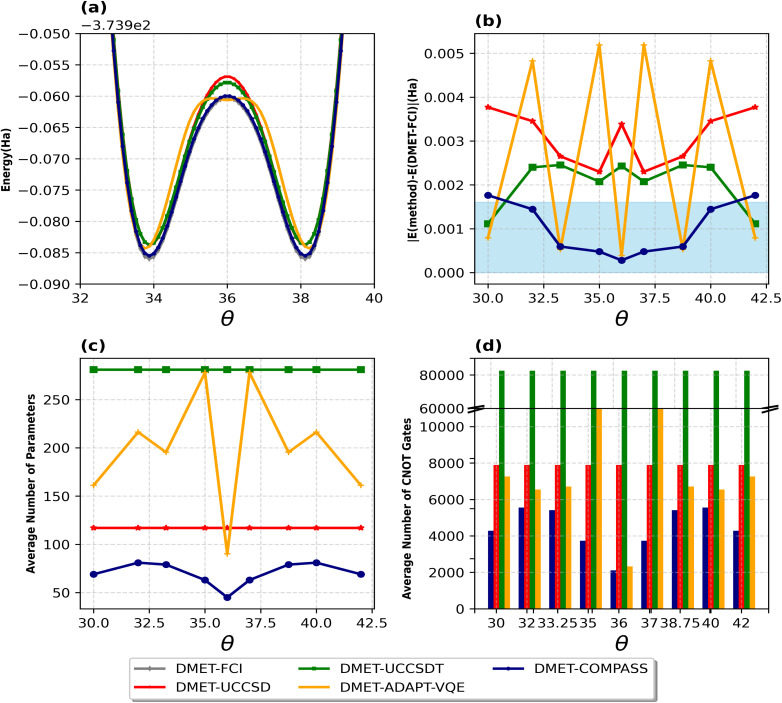
The total energy of various methods is presented in (a), while the energy difference from DMET-FCI is presented in (b). The shaded blue region in (b) indicates chemical accuracy with respect to DMET-FCI. In the second row, the corresponding average number (across all DMET-cycles) of parameters and CNOT gate counts are shown in (c) and (d), respectively, for all methods.

From Fig. 3, it is evident that the accuracy of DMET-COMPASS is significantly superior to that of DMET-UCCSD across the entire range of geometries, and for most configurations, it even achieves better quantitative agreement than DMET-UCCSDT. Unlike DMET-UCCSD and DMET-UCCSDT, where the ansatz structure remains fixed, the ansatz in DMET-COMPASS is dynamically adapted in each DMET cycle. Therefore, we report the number of parameters and CNOT gate counts averaged over all DMET cycles. As shown in Fig. 3, the average parameter count and CNOT gate requirements for DMET-COMPASS are substantially lower than those of both DMET-UCCSD and DMET-UCCSDT, highlighting its superior resource efficiency. Owing to the coupled optimization procedure employed in DMET-COMPASS, the ansatz is reconstructed during successive DMET macro-cycles. However, as shown in SI Section S2, the cumulative number of function evaluations required for ansatz construction and VQE optimization across all DMET cycles along the potential energy surface of circular C_10_ indicates that this coupled optimization framework incurs only a modest computational overhead relative to DMET-UCCSD, while requiring substantially fewer function evaluations than DMET-UCCSDT. These results indicate that the coupled optimization framework employed in DMET-COMPASS does not impose a significant computational overhead to converge the global chemical potential.

**Fig. 4:**
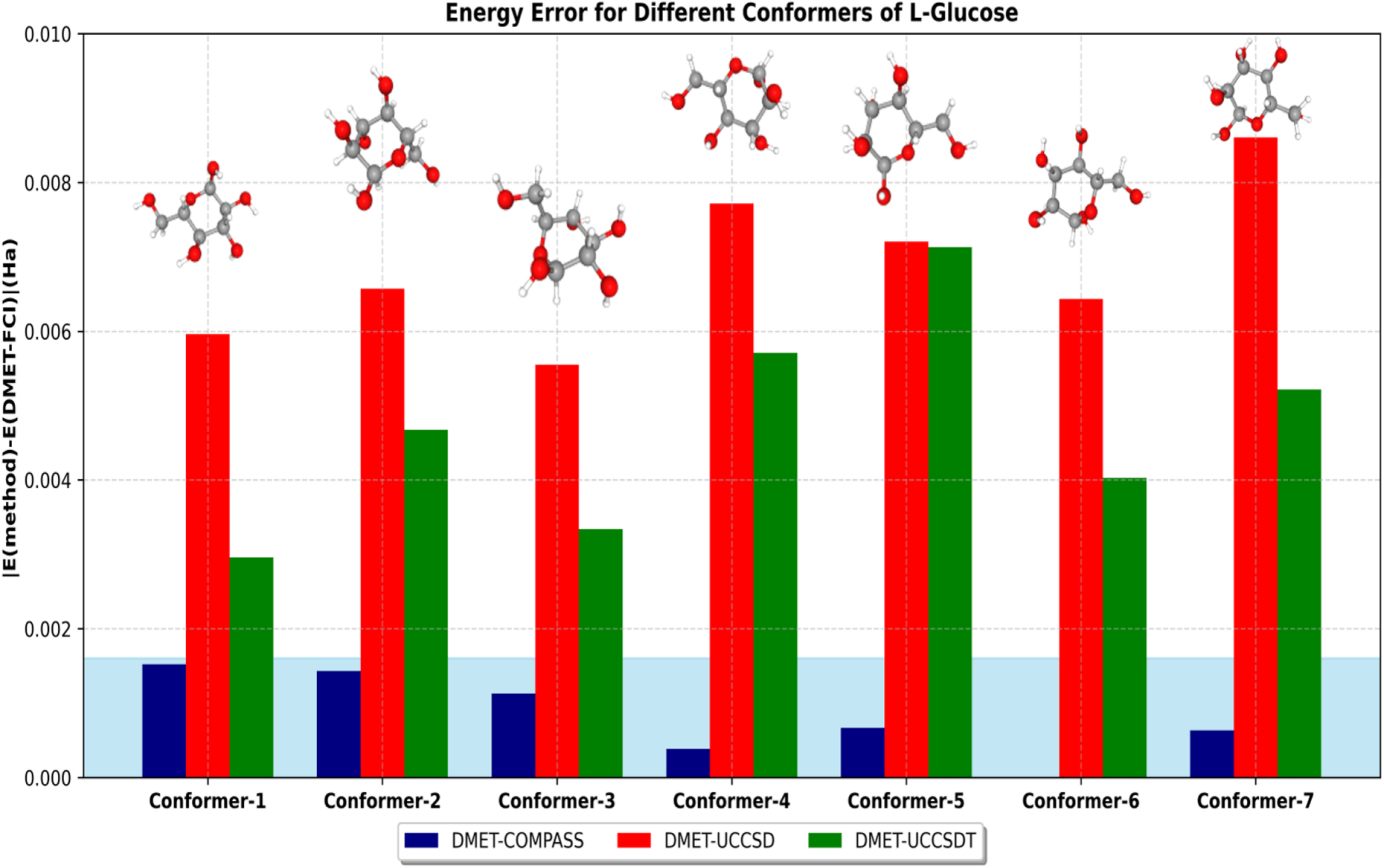
Deviation in energy (in *E*_h_) from DMET-FCI for different conformers of l-glucose is shown for DMET-UCCSD, DMET-UCCSDT and DMET-COMPASS. The shaded light blue region indicates chemical accuracy with respect to DMET-FCI.

We have also evaluated the ground state energies using ADAPT-VQE, a widely used dynamic ansatz construction approach, within the DMET framework using the same active space and fragmentation scheme as employed in DMET-COMPASS. From Fig. 3, it is clear that while DMET-ADAPT-VQE achieves accuracy comparable to DMET-COMPASS for certain geometries, its performance is not consistent across the potential energy profiles. Notably, in all cases, DMET-ADAPT-VQE requires a significantly larger average number of parameters and CNOT gates compared to DMET-COMPASS. This is due to its gradient-based operator selection, which often encounters convergence plateaus, necessitating the inclusion of additional operators to achieve convergence. Furthermore, DMET-ADAPT-VQE typically requires more number of DMET cycles to converge the global chemical potential. However, owing to the dynamic nature, DMET-ADAPT-VQE also requires additional pre-circuit measurements at each DMET macro cycle to identify the dominant operators. A detailed numerical analysis of the measurement requirements (for ansatz construction and those arising from the subsequent VQE optimization) for DMET-COMPASS, DMET-UCCSD, DMET-UCCSDT and DMET-ADAPT-VQE are shown for two different geometries of circular C_10_ in SI Section S3 and Fig. S2. These results demonstrate that although DMET-COMPASS requires additional pre-circuit measurements for ansatz construction, its overall measurement count is only slightly higher than that of DMET-UCCSD and remains substantially lower than those of DMET-UCCSDT and DMET-ADAPT-VQE. These observations collectively demonstrate the effectiveness and efficiency of the proposed DMET-COMPASS approach.

#### 4.2.1. Noisy simulation

All of the results discussed above were obtained under ideal, noise-free simulation conditions. To assess the impact of hardware noise, both the DMET-COMPASS ansatz construction procedure and the subsequent VQE optimization were performed in the presence of a depolarizing noise channel. A detailed comparison of the DMET-COMPASS ansatz structures obtained under noiseless and noisy conditions for two geometries of circular C_10_ has been discussed in SI Section S4 and illustrated in Fig. S3 and S4. In addition, the VQE optimization results for the various methods in the presence of depolarizing noise are also discussed in SI Section S4 and numerically illustrated in Fig. S5, which show a clear improvement in accuracy and a distinctively improved convergence pattern for DMET-COMPASS over the other fixed-structured ansatze.

#### 4.2.2. Comparison with canonical results

The efficiency of DMET-COMPASS in recovering the localized correlation energy has been demonstrated above through direct comparison with DMET-FCI results. However, owing to the highly localized fragmentation employed, the DMET framework is inherently subject to approximation errors. To assess the magnitude of these errors, the DMET results are compared with canonical CCSD and CCSD(T) calculations, as summarized in Table 1. For this study, the system is also partitioned into fragments containing individual carbon atoms. To ensure a fair comparison and keep the computational cost manageable, we froze the core 1s orbital of each carbon atom before constructing the bath orbitals. Consequently, the VQE calculations for each embedding problem are performed on a 16 qubit space. For consistency, the canonical CCSD and CCSD(T) calculations are also carried out using the same frozen-core treatment, with the 1s orbitals of all carbon atoms excluded from the correlation calculation. As evident from Table 1, the DMET energies exhibit significant deviations from the corresponding canonical results. The accuracy of DMET is strongly influenced by the chosen fragmentation strategy, and the optimal fragmentation scheme can vary significantly from one system to another (*vide infra*). Owing to the limitations imposed by the available qubit resources in statevector simulations, we are unable to systematically investigate the dependence of the accuracy on different fragmentation schemes for the system under consideration. However, the effect of fragment size on the quality of the DMET-based results is demonstrated explicitly for the linear H_12_ system, where a clear improvement in accuracy is observed with increasing fragment size.

**Table 1:** The canonical CCSD and CCSD(T) results are compared with those obtained from DMET-FCI, DMET-UCCSD, and DMET-COMPASS. For consistency, the carbon 1s orbitals are frozen in all canonical calculations. Similarly, in all DMET calculations, the embedded subsystem is constructed by freezing the carbon 1s orbitals

C_10_ geometry	RHF (Ha)	CCSD (Ha)	CCSD(T) (Ha)	DMET-FCI (Ha)	DMET-UCCSD (Ha)	DMET-COMPASS (Ha)
30	−373.254645	−373.957381	−373.967539	−374.069877	−374.067899	−374.068101
33.25	−373.462521	−374.245908	−374.267782	−374.400420	−374.397266	−374.397293
36	−373.425442	−374.244896	−374.291175	−374.441903	−374.438701	−374.440033

### 4.3. Ground state energy calculation for different conformers of l-glucose

Carbohydrate chemistry plays a fundamental role in understanding the structure, reactivity, and biological function of sugars, where stereochemistry is a key governing factor. Within this framework, l-glucose, the enantiomer of naturally abundant d-glucose, provides an ideal model to investigate the effects of stereochemistry on molecular properties. In this context, the ground state energy of various conformers of l-glucose may be considered an important factor to determine their relative stability, which we decipher using our newly developed algorithm. The molecular geometries for all conformers are obtained from PubChem.^54^

Similar to the previous case, the full system is partitioned in an atom-wise manner, which significantly reduces the qubit requirement from 144 to at most 20 qubits for the VQE simulations. In this setup, each fragment corresponds to an individual H, C, or O atom. For the C and O fragments, high-level wavefunctions are computed within a (4,4) active space using DMET-COMPASS, DMET-UCCSD, and DMET-UCCSDT approaches. The accuracy of these methods is benchmarked against DMET-FCI results evaluated within the same active space. In the DMET-COMPASS simulation for these test cases, we have used the opposite-spin scattering operator pool as previously defined in Section 4.1.

Although all the DMET-based quantum algorithms predict correct relative energy ordering as DMET-FCI, as shown in Fig. 4, DMET-COMPASS exhibits excellent quantitative agreement. In contrast, the deviations observed for DMET-UCCSD and DMET-UCCSDT relative to DMET-FCI are significantly larger, exceeding the chemical accuracy threshold of 1.6 mHa. A key advantage of DMET-COMPASS is its dynamic nature: during each DMET cycle, the ansatz structure and length dynamically change in response to variations in the global chemical potential. This is in contrast to DMET-UCCSD and DMET-UCCSDT, where the ansatz remains fixed throughout the DMET iterations. For the different l-glucose conformers, the maximum number of parameters (CNOT gates) required for the high-level wavefunction calculations is 24 (1016 CNOT gates) in the case of DMET-COMPASS. In comparison, DMET-UCCSD and DMET-UCCSDT require 26 (1126 CNOT gates) and 34 (3870 CNOT gates), respectively. These results clearly demonstrate that DMET-COMPASS is more efficient in capturing localized correlation effects within each embedded subsystem while utilizing significantly fewer quantum resources than DMET-UCCSD and DMET-UCCSDT.

**Fig. 5:**
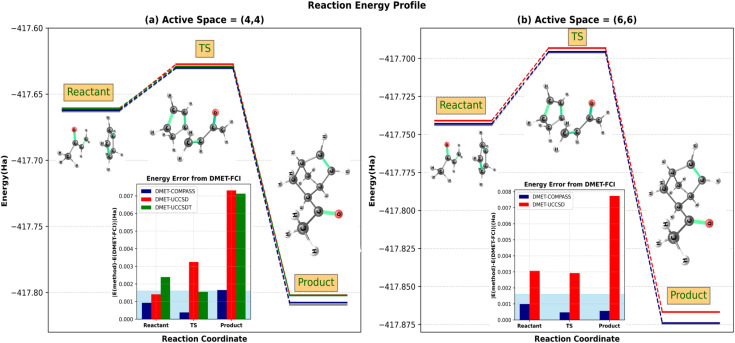
Reaction energy profile of the (4 + 2) cycloaddition reaction between cyclopentadiene and methyl vinyl ketone is presented for DMET-UCCSD, DMET-UCCSDT and DMET-COMPASS. The two different plots (a) and (b) indicate the different active space dimensions ((4,4) and (6,6), respectively) for the high-level wavefunction calculation of the embedded subsystems (for C and O atom fragments). The inset plots indicate the corresponding energy deviation from DMET-FCI. The blue shaded region indicates chemical accuracy with respect to DMET-FCI.

### 4.4. Reaction energy profile of the Diels–Alder reaction

In this section, we investigate the reaction energy profile of the Diels–Alder reaction, one of the most widely utilized and extensively studied transformations in organic chemistry. As a representative case, we consider the cycloaddition between methyl vinyl ketone (MVK) and cyclopentadiene (CP). The optimized geometries of the reactant, transition state, and product employed in this work were obtained from prior DMET-D3 calculations.^55^ In this benchmarking study, we divided the whole system (reactant, intermediate and product) into atom-wise fragments. Each fragment comprises individual atoms (H, C, or O), enabling a significant reduction in the effective system size. Through this fragmentation scheme, the original problem size of 124 qubits is reduced to embedded subsystems requiring at most 20 qubits.

Within each embedded subsystem (except H fragment), the high-level wavefunction is calculated *via* considering active space dimensions of (4,4) and (6,6). In both situations, we have determined the high-level wavefunction *via* UCCSD, UCCSDT and COMPASS strategies and compared the results with DMET-FCI within the corresponding active space dimensions. In the case of DMET-COMPASS, we have used the partial-pairing scattering operator pool.

From Fig. 5, it is evident that for reactants, intermediates, and products, across both active space dimensions, the accuracy of DMET-COMPASS is significantly superior to that of DMET-UCCSD and DMET-UCCSDT. This indicates that DMET-COMPASS is more effective in capturing localized electron correlation compared to the other two approaches. In particular, in the case of the product, where two new σ-bonds are formed from the breaking of two π-bonds during the Diels–Alder reaction, DMET-COMPASS demonstrates a notably improved description of strong correlation effects. Moreover, in all cases, the DMET-COMPASS energies reach chemical accuracy with respect to DMET-FCI calculated in their respective active space dimensions. Importantly, for the product, which exhibits strong correlation, the high-level wavefunction calculations performed in a (6,6) active space across the C and O fragments demonstrate a substantial reduction in quantum resources. On average (considering all C and O fragments over all DMET cycles), DMET-COMPASS requires 4029 CNOT gates. In comparison, the corresponding DMET-UCCSD calculations require 7893 CNOT gates, while DMET-UCCSDT demands 84 477 CNOT gates. This clearly highlights the superior resource efficiency of DMET-COMPASS.

**Fig. 6:**
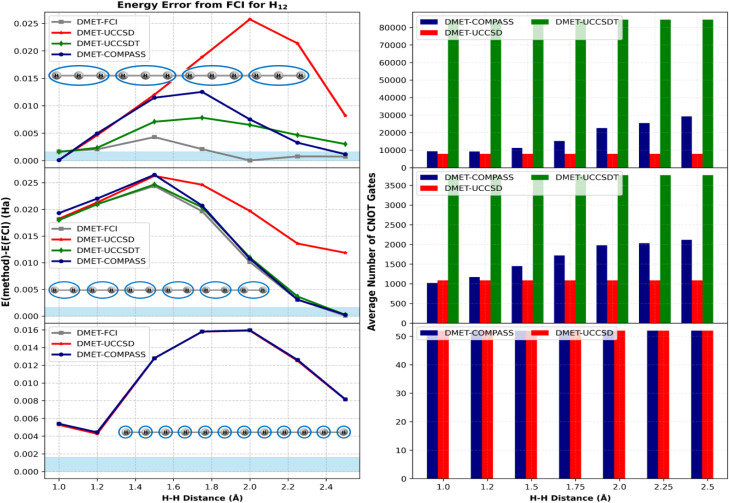
Energy errors with respect to canonical FCI are presented for different methods and fragmentation schemes for the linear H_12_ system as a function of the H–H bond length. The shaded blue region denotes chemical accuracy relative to FCI. The corresponding average number of CNOT gates (averaged over all fragments and DMET cycles) is shown in the bar plots.

### 4.5. Effects of different fragmentation schemes: accuracy *vs.* quantum efficiency

In all the previous test cases, we constructed the embedded subsystems by partitioning the full system in an atom-wise manner and benchmarked the results of our approach against DMET-FCI. However, it is equally important to assess how the various DMET based strategies perform with respect to the exact full system FCI calculation and how this deviation depends on the chosen fragmentation scheme. To this end, we consider a moderately sized, strongly correlated system, the linear H_12_ chain, for which the exact FCI energy can be computed within the STO-3G basis. In this system, all H–H bond distances are kept identical, and the potential energy surface is explored by varying the bond length from 1.0 Å to 2.5 Å. We adopt three different fragmentation schemes: in the first case, the system is divided into 12 individual H atoms; in the second case, it is partitioned into 6 fragments, each consisting of an H_2_ unit; and in the third case, the system is divided into 4 fragments, each containing an H_3_ unit. For all fragmentation schemes, we compute the ground state energies using DMET-FCI, DMET-COMPASS, and DMET-UCCSD, and compare the results against the canonical exact FCI energy of the full system.

It is important to note that in the first fragmentation scheme, where linear H_12_ is partitioned into 12 individual H atoms, each embedded subsystem contains 2 electrons in 4 spin–orbitals. In this case, only one double excitation is possible, with no scope for triple or higher-order excitations. Consequently, within the DMET-COMPASS framework, no scattering operators are involved. As a result, the performances of DMET-COMPASS and DMET-UCCSD are expected to be identical, which is also reflected in Fig. 6. However, the results obtained from DMET-based methods throughout the potential energy surface remain relatively poor for this fragmentation scheme in general. In the second fragmentation scheme, where each fragment consists of one H–H unit, the accuracy of DMET-FCI improves significantly, particularly in the strongly correlated regime. However, this improvement is not uniform across the entire potential energy surface. In this case, both DMET-COMPASS and DMET-UCCSDT exhibit comparable accuracy to the DMET-FCI results throughout the surface, whereas DMET-UCCSD shows noticeable deviations, especially at larger bond distances, as shown in Fig. 6. The average number of CNOT gates required for the DMET-COMPASS ansatz is slightly higher than that of DMET-UCCSD, yet remains substantially lower than that of DMET-UCCSDT. Finally, in the third fragmentation scheme, where each fragment comprises an H–H–H unit, the accuracy of DMET-FCI improves considerably across the entire potential energy surface, approaching chemical accuracy in most regions. While DMET-UCCSD performs poorly throughout, DMET-COMPASS demonstrates strong quantitative agreement with DMET-FCI, particularly in the strongly correlated regime. Although minor deviations are observed at intermediate bond distances, its overall performance is significantly better than DMET-UCCSD. In terms of quantum resources, the average CNOT gate count for DMET-COMPASS is higher than that of DMET-UCCSD but remains substantially lower than that required for DMET-UCCSDT. The accuracy and resource efficiency of the DMET-COMPASS framework can be further enhanced by appropriately modifying the CSOs involved in the scattering operators (currently, only the HOMO and LUMO are taken as CSO across all fragmentation schemes) and by systematically eliminating those scatterers that introduce redundant higher-order contributions. Such refinements help to reduce unnecessary circuit complexity while retaining the essential correlation effects. It is particularly important to note that in the final fragmentation scheme, where each embedded subsystem requires a 12 qubit VQE simulation within the DMET framework, it is quite possible to reach within the chemical accuracy with respect to the canonical FCI energy. In contrast, a full simulation of the H_12_ system on a quantum computer would require 24 qubits, which is twice the number needed in the DMET-based approach. This clearly demonstrates the superior resource efficiency of DMET methodologies for large-scale molecular simulations. In this case, it is observed that increasing the fragment size tends to improve the accuracy of DMET-based methods. However, this trend is not universal, and the optimal fragmentation strategy is inherently system dependent.

**Fig. 7:**
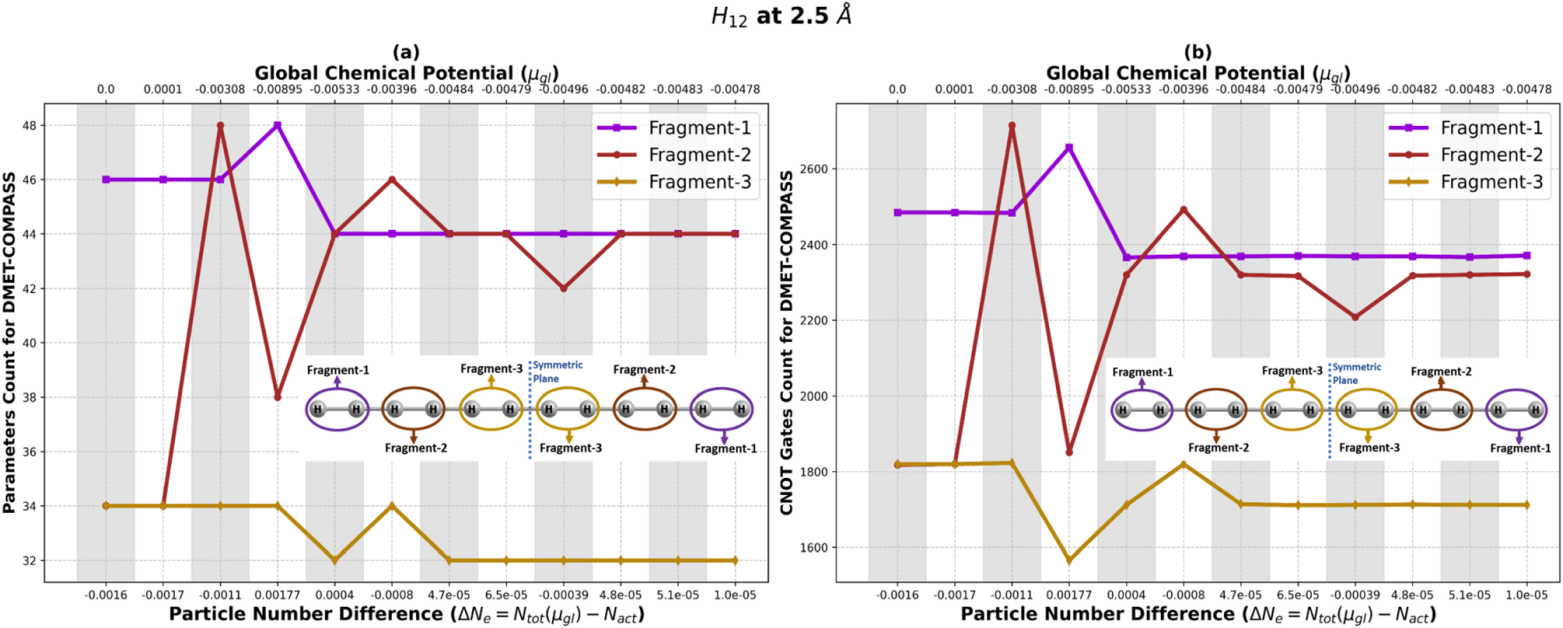
Variation in the (a) number of parameters and (b) CNOT gate count for the DMET-COMPASS ansatz as functions of the global chemical potential at each stage of the DMET optimization cycle are presented for different inequivalent fragments of the linear H_12_ system. The H–H bond length is fixed at 2.5 Å. At each cycle, with the variation of global chemical potential, the convergence of the number of particles is also presented.

Beyond the total energy, various molecular properties of the full system can be evaluated from the reduced density matrices of the embedding subsystems. A comparison between the electronic dipole moment obtained using DMET-COMPASS and those obtained from canonical FCI calculations for linear H_12_ is shown in the SI (Section S1).

### 4.6. Adaptability of the DMET-COMPASS ansatz with respect to the global chemical potential

In this subsection, we demonstrate how the DMET-COMPASS ansatz corresponding to each embedded subsystem dynamically adapts with the variation of the global chemical potential (*µ*_gl_) across successive DMET macro cycles. For this purpose, we consider the linear H_12_ system at an H–H bond length of 2.5 Å, where the full system is partitioned into six fragments, each comprising an H–H unit. As this is a symmetric system with respect to the plane containing the point of inversion, three different types of fragment units are present.

For each of these fragment types, we analyze how the DMET-COMPASS ansatz length changes over different DMET macro-cycles. In Fig. 7, we present the variation of the number of variational parameters along with the corresponding CNOT gate counts as functions of the global chemical potential and the particle number difference at each macro cycle. It is evident from Fig. 7 that different fragments require different ansatz sizes within the DMET-COMPASS framework. More importantly, even for a given fragment, the ansatz length varies from one DMET macro cycle to another. This behavior highlights that, as the global chemical potential is updated at each macro-cycle, the corresponding embedded Hamiltonians are modified. As a result, the DMET-COMPASS ansatz auto-adjusts to accurately capture the correlation effects. Therefore, DMET-COMPASS inherently involves a coupled optimization of the global chemical potential and the ansatz structure. This coupling provides enhanced flexibility, enabling the ansatz to adapt consistently with the changes in the global chemical potential. Furthermore, Fig. 7 shows that the variation in ansatz length is more pronounced during the initial DMET cycles and gradually stabilizes as convergence is approached. It is also important to note that, although two different fragments may require the same number of parameters (as shown in Fig. 7(a)), their corresponding CNOT gate counts (Fig. 7(b)) can differ. This indicates that distinct operator sets are dynamically picked up to form the ansatz in different fragments. While in most cases DMET-COMPASS and DMET-UCCSD take a similar number of DMET cycles to converge the global chemical potential, there may exist some cases (like the one presented in Fig. 7) where DMET-COMPASS takes more cycles. However, in such cases, DMET-COMPASS keeps adjusting the ansatz length and ordering for each embedded subsystem over more DMET cycles to arrive at significantly better energy values than DMET-UCCSD.

## 5. Conclusions

Due to the limitation of fault-tolerant qubits and the limited fidelity of quantum gates, performing accurate, realistic chemical simulations remains beyond the reach of present day quantum hardware. To address this challenge, partition-based embedding approaches provide an effective route by reducing the size of the full system while preserving accuracy through the evaluation of localized correlation energies within individual fragments. The overall performance of such embedding frameworks can be enhanced in two primary ways: first, by refining the embedding scheme to achieve a more accurate representation of the embedded subsystems, and second, by improving the quality of the correlated high-level wavefunction so that it can more effectively capture the localized correlation effects and use the high-level wavefunction to self-consistently update the embedding Hamiltonian.

In this work, we employ the latter strategy within the DMET framework to enhance the description of the ground state of chemically relevant systems and processes. The procedure relies on capturing the localized correlation energy *via* the construction of dynamic high-level wavefunctions for multiple embedding problems through a systematic partitioning of the full system. For each embedded Hamiltonian, a dynamic ansatz is generated *via* a commutativity-based local operator selection and an energy-driven operator screening procedure tailored to the corresponding subsystem. During every DMET iteration, these subsystem specific high-level wavefunctions are used to update the global chemical potential, which in turn refines the embedding Hamiltonians. Unlike any statically structured ansatz for the construction of the high-level wavefunction, working with a dynamic ansatz in the DMET framework warrants the coupled optimization of the global chemical potential (thereby the embedding Hamiltonian) and the ansatz structure for each of the embedding problems. This introduces additional flexibility in the ansatz design and operator ordering. However, the measurement overhead associated with ansatz construction at each DMET cycle could potentially be reduced by employing a threshold criterion on the variation of the global chemical potential. Under such a scheme, ansatz reconstruction would only be triggered when the change in the chemical potential surpasses a specified threshold. Our numerical applications involving strong correlation suggest that such a strategy of dynamic ansatz construction within an embedding problem attains very high accuracy for important chemical problems over more conventional ansatze with significantly lower quantum gate utilization.

Furthermore, we investigate the improvement in the total system energy within the DMET framework as a function of fragment size for a strongly correlated linear H_12_ chain. The results highlight the critical role of the fragmentation scheme in capturing correlation effects, showing that increasing the fragment size systematically enhances the recovered correlation energy and approaches chemical accuracy relative to the full system. Moreover, it is demonstrated how the ansatz adjusts itself for each embedding problem at each step of the DMET cycle as the global chemical potential (or equivalently embedding Hamiltonian) is updated, enabling an accurate representation of correlation effects while maintaining low quantum resource requirements. However, the effectiveness of a given partitioning scheme remains system dependent, emphasizing the importance of designing an optimal fragment-bath subsystem, which is an open direction in embedding methodologies. Additional improvements in both accuracy and resource efficiency of the high-level solver in DMET-COMPASS can be achieved through careful tuning of screening thresholds, elimination of redundant higher-order excitations, and reduction of measurement overhead *via* Pauli term grouping in embedded Hamiltonians.^56–58^ For practical implementations on quantum hardware, integration of the high-level solver with proper quantum error mitigation techniques is necessary to further improve the accuracy.^59–61^ Furthermore, generalizing the scheme to treat open-shell systems as they appear in transition-metal catalysis or bond-breaking processes would be an extremely important avenue to pursue in the near future. Overall, the current development suggests that the combination of dynamic, resource-efficient ansatz construction within an advanced embedding framework presents a promising route toward accurate and scalable simulations of strongly correlated, classically intractable chemical systems.

## Author contributions

Conceptualization: Dibyendu Mondal and Rahul Maitra; methodology: Dibyendu Mondal and Rahul Maitra; investigation: Dibyendu Mondal and Ashish Kumar Patra; formal analysis: Dibyendu Mondal, Ashish Kumar Patra, and Rahul Maitra; data curation: Dibyendu Mondal; writing – original draft: Dibyendu Mondal and Rahul Maitra; writing – review and editing: Dibyendu Mondal, Ashish Kumar Patra, and Rahul Maitra; software: Dibyendu Mondal and Ashish Kumar Patra; visualization: Dibyendu Mondal and Ashish Kumar Patra; supervision: Rahul Maitra; funding acquisition: Rahul Maitra.

## Conflicts of interest

The authors have no conflicts of interest to disclose.

## Supplementary Material

SC-OLF-D6SC03132C-s001

## Data Availability

The numerical data that support the findings of this study are available from the corresponding author upon reasonable request. Additional numerical results, including electronic dipole moment calculations, measurement counts, and noisy simulation studies, are available in the supplementary information (SI). Supplementary information is available. See DOI: https://doi.org/10.1039/d6sc03132c.
