# Supplementary material for: Advancing practical quantum embedding simulations via operator commutativity-based state preparation for complex chemical systems
Source: Chem Sci. 2026 Aug 4;17(39):18685–700. doi: 10.1039/d6sc03132c (PMC13482333; doi:10.1039/d6sc03132c)
Supplement: Supplementary file 1 [file SC-017-D6SC03132C-s001.pdf]

Supplementary Information (SI) for Chemical Science.
This journal is © The Royal Society of Chemistry 2026

Electronic Supplementary Information: Advancing Practical Quantum Embedding Simulations via Operator Commutativity Based State Preparation for Complex Chemical Systems

Dibyendu Mondal

Department of Chemistry, Indian Institute of Technology Bombay
Powai, Mumbai 400076, India

Ashish Kumar Patra

Qclairvoyance Quantum Labs, Secunderabad, TG 500094, India

Rahul Maitra*

Department of Chemistry, Indian Institute of Technology Bombay
Powai, Mumbai 400076, India and
Centre of Excellence in Quantum Information,
Computing, Science & Technology,
Indian Institute of Technology Bombay,
Powai, Mumbai 400076, India

S1. MOLECULAR PROPERTIES CALCULATION: ELECTRONIC DIPOLE MOMENT DETERMINATION

In addition to the total energy of the full system, various molecular properties can also be evaluated from the correlated description of embedding systems in DMET. In each DMET cycle, a correlated wavefunction is obtained for every embedding subsystem. From these correlated wavefunctions, the corresponding energies, one-particle reduced density matrices (1-RDMs), and two-particle reduced density matrices (2-RDMs) are evaluated. These reduced density matrices are often used to compute a variety of molecular properties, including electronic dipole moments, bond orders, charge and spin populations, and magnetic properties. To demonstrate this capability, here we calculated the electronic dipole moment for a few selected $H-H$ bond distances of the linear (H_{12}) chain. In this study, the system was partitioned into six (H_2) fragments, and both the energies and electronic dipole moments obtained using DMET-COMPASS were compared against the corresponding canonical FCI results for the full system.

The calculation of the electronic dipole moment of the entire system requires the construction of a global 1-RDM. To achieve this, the embedded 1-RDMs obtained from the correlated wavefunction of each subsystem are first transformed from the embedding molecular orbital basis to the localized orbital basis using the embedding SCF molecular orbital coefficients. Subsequently, the global 1-RDM in the localized orbital basis is assembled by projecting and combining only the fragment orbital blocks from the embedding 1-RDMs. The resulting global density matrix is then transformed from the localized orbital basis to the atomic

* rmaitra@chem.iitb.ac.in

orbital (AO) basis, (γ_{pq}), using the Meta- Lowdin localized molecular orbital coefficient matrix. The one-electron dipole integrals, ($\langle\chi_p|O|\chi_q\rangle$) ($O = x, y, z$), are imported from PySCF in the AO basis. The electronic contribution to the dipole moment is then computed by contracting these dipole integrals with the AO density matrix according to $\mu_O = -\sum_{pq}\gamma_{pq}\langle\chi_p|O|\chi_q\rangle$.

From the Table S1, it is evident that as the energy difference between DMET-COMPASS and the full- system canonical FCI decreases, the corresponding electronic dipole moments show increasingly better agreement with the canonical electronic dipole moment values.

H-H Distance (Å)	E_{FCI} (Hartree)	$E_{DMET-COMPASS}$ (Hartree)	μ_{FCI} (a.u.)	$\mu_{DMET-COMPASS}$ (a.u.)
**1.0**	**-6.452815**	**-6.433505**	**-124.721924**	**-124.599774**
**1.5**	**-5.995582**	**-5.969096**	**-187.082886**	**-187.072379**
**2.0**	**-5.695801**	**-5.685031**	**-249.443848**	**-249.437684**
**2.5**	**-5.617127**	**-5.616879**	**-311.804810**	**-311.805457**

TABLE S1: Total energy and electronic dipole moment are computed by full canonical FCI and DMET-COMPASS method for the H_{12} linear chain (STO-3G basis) at four different $H-H$ bond distances.

S2. STABILITY OF COUPLED OPTIMIZATION

In this section, we examine whether the repeated ansatz construction performed at each DMET macro-cycle introduces any additional difficulty in converging the global chemical potential. In contrast to DMET-UCCSD and DMET-UCCSDT, where a fixed ansatz is employed throughout the DMET procedure, DMET-COMPASS utilizes a coupled optimization framework in which the ansatz is reconstructed during successive DMET cycles. Although in the strongly correlated regime, we have observed the changes in DMET-COMPASS ansatz structure, particularly in the initial few DMET cycles as shown in Fig. 7 in the main text for linear H_{12}, this however does not render in any convergence difficulty for successive DMET macro-cycles. This observation is consistent across various systems. However, the coupled optimization strategy in DMET-COMPASS introduces only a modest additional computational cost (in terms of pre-circuit measurement to construct the ansatz) relative to DMET-UCCSD and DMET-UCCSDT. To quantify this overhead, Fig. S1 presents the cumulative number of function evaluations required for ansatz construction and VQE optimization across all DMET cycles along the potential energy surface of circular C_{10} for DMET-COMPASS, DMET-UCCSD, and DMET-UCCSDT. As evident from the figure, the total number of function evaluations required by DMET-COMPASS is only slightly higher than that of DMET-UCCSD, but it is substantially lower than that of DMET-UCCSDT. These results indicate that the coupled optimization framework employed in DMET-COMPASS does not impose a significant computational overhead to converge the global chemical potential.

S3. TOTAL MEASUREMENT REQUIREMENTS FOR EACH ANSATZ

In this section, we compare the total measurement count associated with different ansatze, including both the measurements required for ansatz construction (for dynamic ansatze like COMPASS- and ADAPT-VQE) and those arising from the subsequent

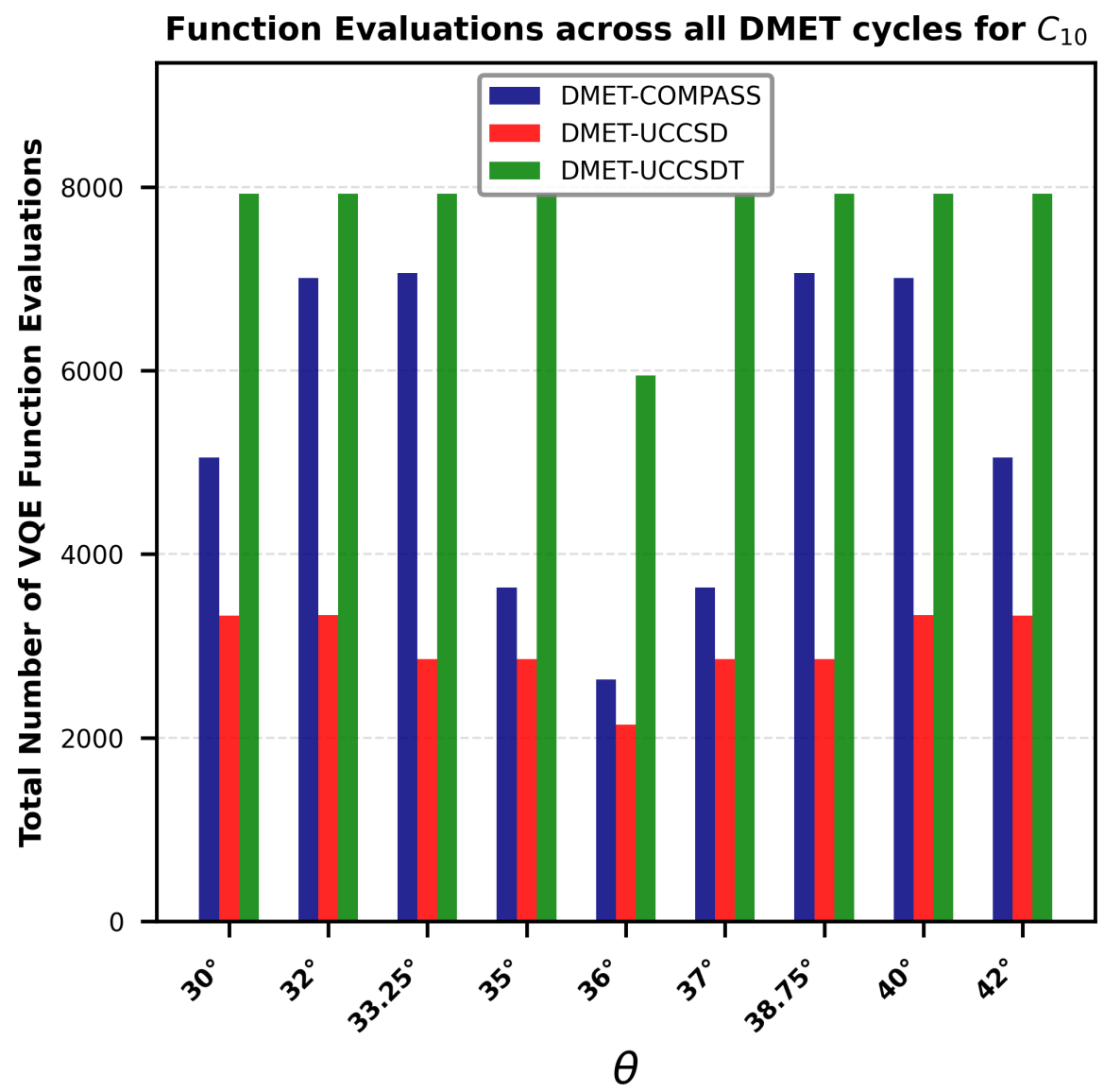

Fig. S1: **The cumulative number of VQE function evaluations, including the evaluations associated with ansatz preparation, across all DMET cycles is shown for DMET-COMPASS, DMET-UCCSD, and DMET-UCCSDT along the potential energy surface of circular C_{10}.**

VQE optimization, accumulated over all DMET self-consistency cycles for two geometries of circular C_{10}. The full system is divided into ten carbon atoms fragment (which are equivalent in nature) and the correlation calculation is carried out in the (6,6) active space of each embedding subsystem. Owing to their dynamic nature, DMET-COMPASS and DMET-ADAPT-VQE require additional pre-circuit measurements at each DMET macro-cycle to identify the dominant operators that are incorporated into the final ansatz. In contrast, DMET-UCCSD and DMET-UCCSDT employ fixed operator pools and therefore do not require any pre-circuit measurements for ansatz construction. Consequently, the total measurement cost of adaptive approaches consists of contributions from both operator-selection measurements and VQE optimization, whereas for DMET-UCCSD and DMET-UCCSDT it arises solely from the VQE optimization procedure. However, the ansatz construction protocol in DMET-COMPASS involves only a series of one- and two-parameter VQE micro-cycles, each of which is associated with a relatively small measurement overhead. While circuit depth is an important metric for assessing the practicality of quantum algorithms, the total measurement cost is equally important in determining the overall computational efficiency. In the DMET-COMPASS protocol, the measurement complexity associated with the one- and two-parameter VQE optimizations scales as $O(N^4)$ and $O(2N^4)$, respectively, since the Hamiltonian contains at most two-body terms[1, 2]. Here, N is the number of qubits (spinorbitals) of the system. In contrast, the DMET-UCCSD ansatz does not require any pre-circuit measurements for ansatz construction. However, the subsequent VQE optimization of the UCCSD ansatz still requires a substantial number of measurements to evaluate the energy and optimize the parameters. Contrarily, ADAPT-VQE relies on gradient evaluations to select the dominant operators. The gradient evaluation of each operator requires the computation of a commutator, resulting in a measurement cost that typically scales as $O(N^6)$, comparable to the computational cost associated with evaluating the three-particle reduced density matrix (3-RDM)[3]. In addition, ADAPT-VQE requires repeated VQE optimizations at every ansatz growth cycle, introducing further measurement overhead throughout the calculation. To provide a more quantitative comparison of these costs, a detailed analysis

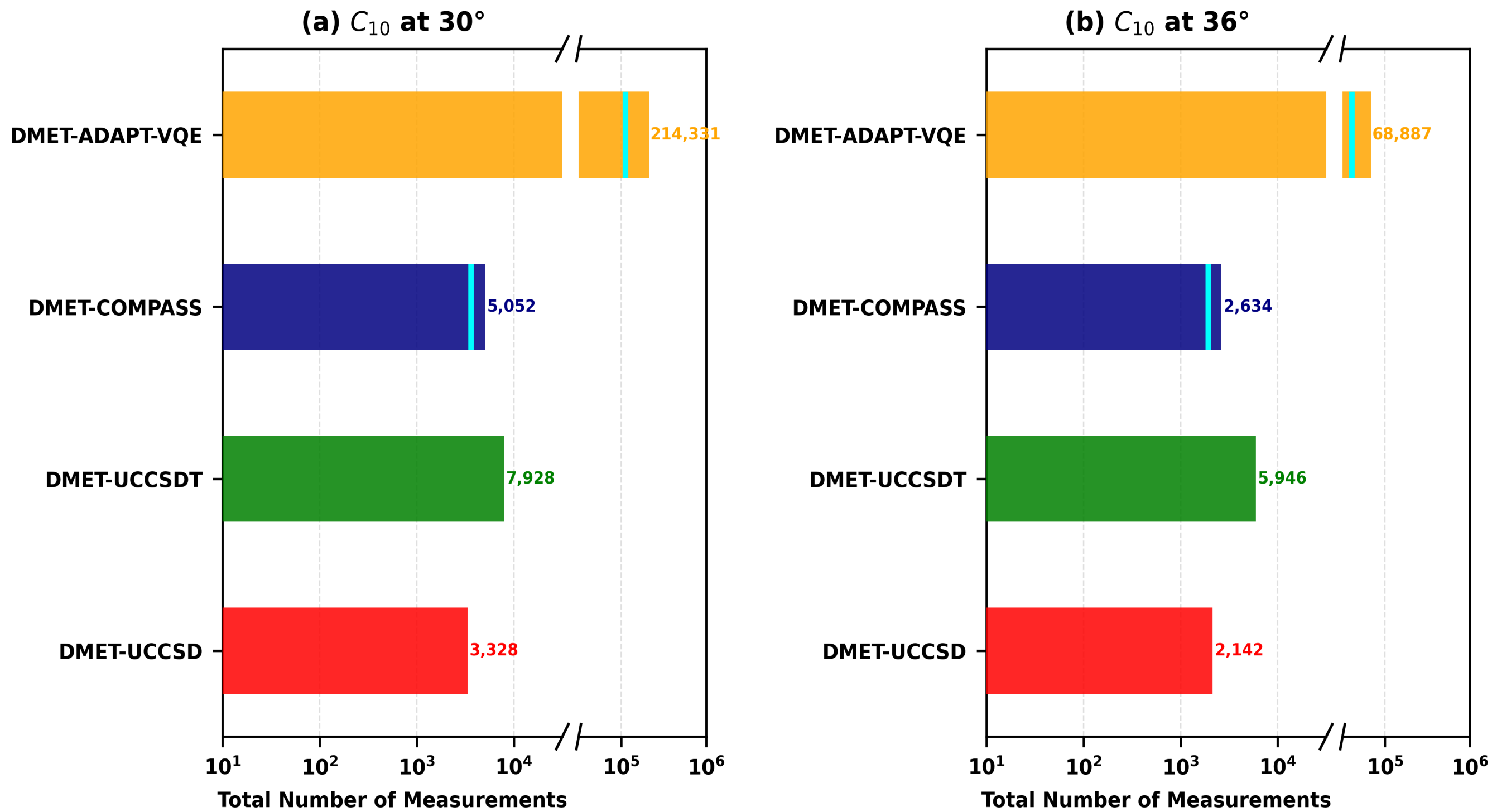

Fig. S2: **The total number of measurements (ansatz construction + VQE optimization) accumulated over all DMET cycles is shown for the different methods at two geometries of circular C_{10}. For DMET-COMPASS and DMET-ADAPT-VQE, the cyan virtical line indicates the number of measurements required for ansatz construction, summed over all DMET cycles.**

of the measurement requirements for DMET-COMPASS, DMET-UCCSD, and DMET-ADAPT-VQE is shown inoverhead Fig. S2.

From Fig. S2, it is evident that the total number of measurements required across all DMET cycles until convergence for DMET-COMPASS is only slightly higher than that of DMET-UCCSD, while it is substantially lower than those required for DMET-UCCSDT and DMET-ADAPT-VQE. Importantly, the measurements associated with ansatz construction throughout all DMET cycles are approximately an order of magnitude fewer compared to DMET-ADAPT-VQE. Furthermore, the figure clearly demonstrates that the significant reduction in the number of operators in COMPASS-VQE leads to more efficient VQE optimization. Consequently, the optimization of the full DMET-COMPASS ansatz requires considerably fewer measurements to reach convergence than both DMET-UCCSD and DMET-UCCSDT.

S4. SIMULATION UNDER SYNTHETIC DEPOLARIZING NOISE CHANNEL

In this section, we examine the impact of noise on the ansatz construction of each embedding system and the subsequent VQE optimization. For the converged global chemical potential obtained from noiseless DMET simulations, we introduced synthetic all qubit depolarizing noise, which represents one of the dominant noise sources in current NISQ devices. In presence of noise the DMET-COMPASS ansatz is constructed and optimized. We considered one- and two-qubit gate error probabilities of (10^{-3})

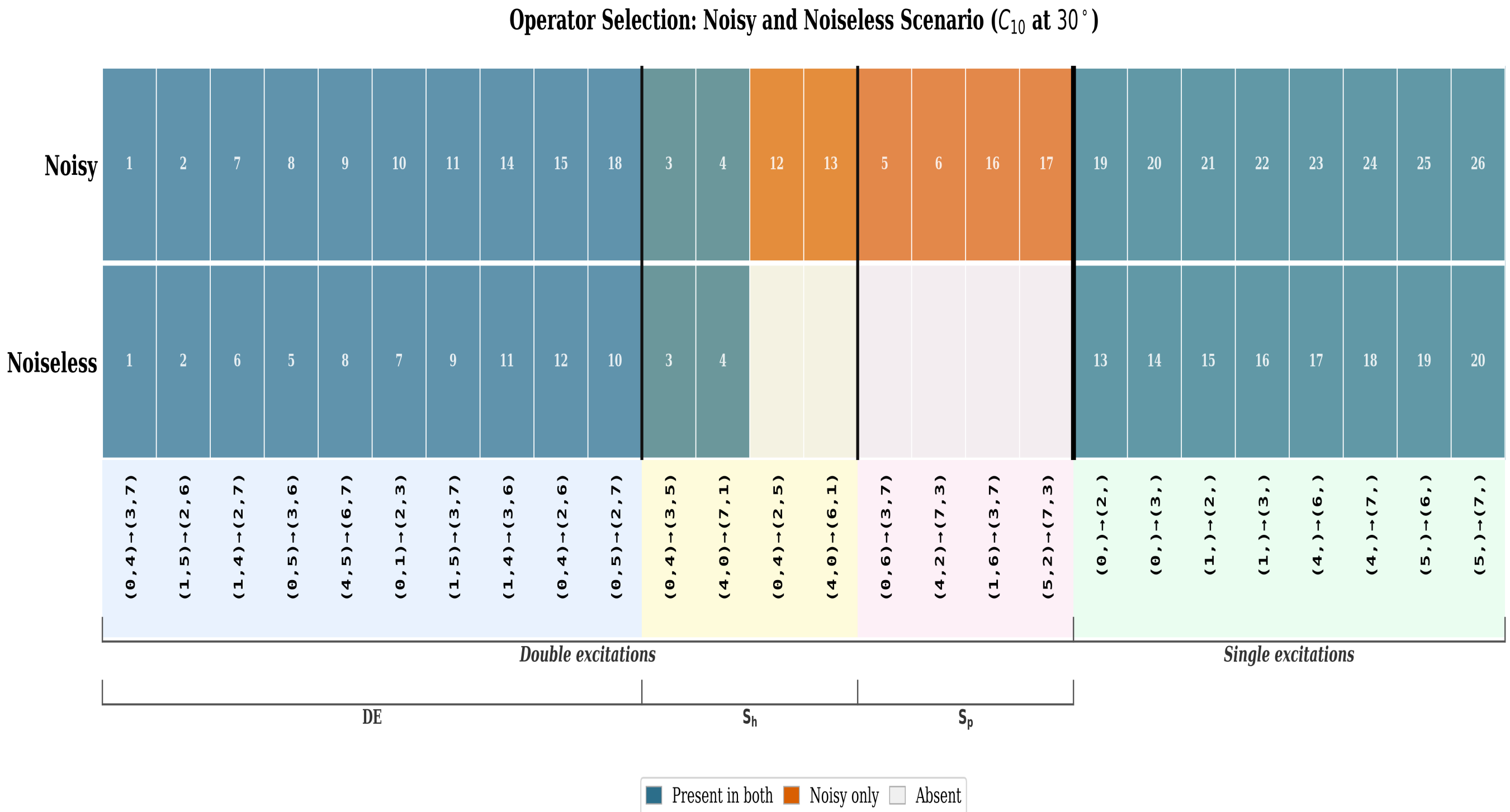

Fig. S3: **The structures of the DMET-COMPASS ansatz constructed under both the noiseless and depolarizing noise settings are shown. The one-body and two-body excitation operators, along with the scatterers, are explicitly indicated. The numbers within the colored blocks denote the positions of the corresponding operators in the ansatz sequence relative to the reference state. The operators corresponding to the un-numbered blocks that appear under noiseless setting do not appear in the ansatz.**

and (10^{-2}), respectively. To assess the impact of noise, we selected two different geometries of the circular (C_{10}) molecule, partitioned into ten one-carbon atom fragments. Here one must note that all the fragments are equivalent. The VQE calculations were carried out within the (4,4) active space of a representative embedding system. We first analyze the effect of depolarizing noise on the DMET-COMPASS ansatz structures relative to the noiseless case. We also report the VQE convergence results with the constructed ansatz with the above mentioned synthetic noise backend in two different cases: (1) the DMET-COMPASS ansatz is constructed with the one- and two-body excitation operators and scattering operators selected under noiseless condition (defined as DMET-COMPASS (noiseless selection), and (2) the ansatz is constructed with the one- and two-body excitation operators and scattering operators selected under noisy conditions (defined as DMET-COMPASS (noisy selection).

From the Fig. S3 and Fig. S4, it is clear that for both molecular geometries, the important two-body excitation operators are identified correctly even in the presence of noise, although their relative ordering changes slightly. In contrast, because a very relaxed threshold of 10^{-7} was used for the scatterer selection, a large number of non-significant scattering operators were selected in both cases. Under such conditions, noise dominates over the weak correlation signals associated with these operators. Consequently, a more restrictive threshold, together with appropriate error mitigation strategies, would be beneficial to improve operator selection. It is important to note that this variation in ansatz structure in the presence of noise is expected for any measurement based dynamic ansatz construction strategy (like gradient based operator selection in ADAPT- VQE is also severely impacted by hardware noise as shown previously[4]. However, this issue is expected to diminish as quantum hardware

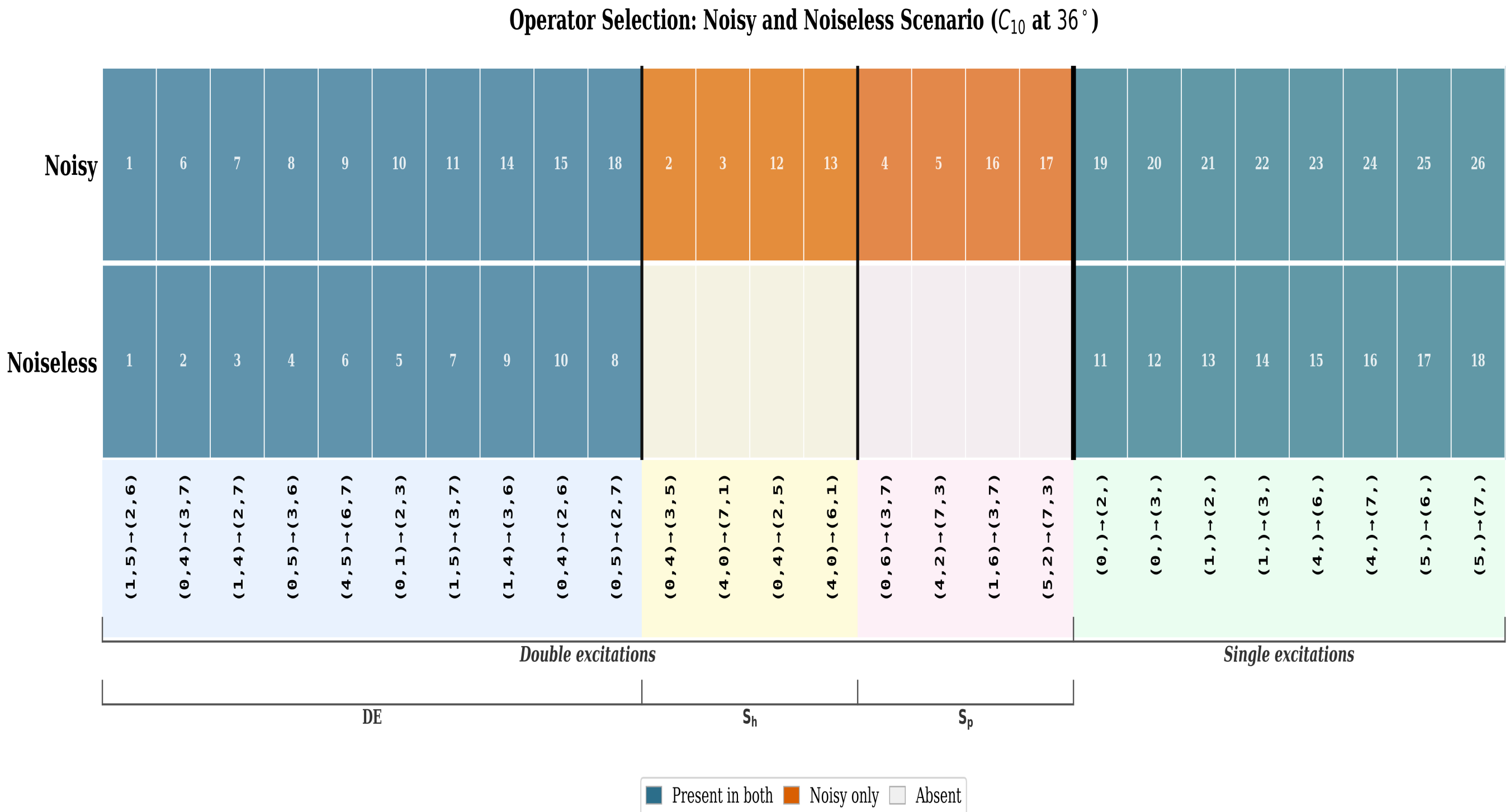

Fig. S4: **The structures of the DMET-COMPASS ansatz constructed under both the noiseless and depolarizing noise settings are shown. The one-body and two-body excitation operators, along with the scatterers, are explicitly indicated. The numbers within the colored blocks denote the positions of the corresponding operators in the ansatz sequence relative to the reference state. The operators corresponding to the un-numbered blocks that appear under noiseless setting do not appear in the ansatz.**

continues to improve.

We further compared the VQE optimization of DMET-COMPASS, DMET-UCCSD, and DMET-UCCSDT in presence of noise. For each ansatz, five independent optimization runs were performed, and the average energy, along with the corresponding standard deviations, is plotted throughout the VQE optimization process. The COBYLA optimizer was employed for all noisy simulations. As shown in Fig. S5, the DMET-UCCSDT ansatz exhibits the largest energy fluctuations because it requires the highest number of quantum gates. Moreover, no clear convergence behavior is observed in this case. For DMET-COMPASS, the presence of noise during ansatz construction (case 2 above) leads to the inclusion of several unimportant scattering operators, increasing the overall circuit depth beyond that of DMET-UCCSD. As a result, the optimized noisy energy of DMET-COMPASS is slightly higher than that of DMET-UCCSD. However, when comparing the initial and final VQE energies, DMET-COMPASS still demonstrates a clear convergence trend despite its larger circuit depth which implies it can capture the weak correlation signal better. To further investigate the effect of operator selection, we performed an additional experiment in which the DMET-COMPASS ansatz was constructed under noiseless conditions (case 1 above) and subsequently optimized in the presence of noise. This allows the ansatz to retain the correct operator set while still capturing the impact of noisy optimization. As illustrated in Fig. S5, in this case DMET-COMPASS achieves approximately an order of magnitude improvement in accuracy compared to all previous noisy selection scenarios, demonstrating its strong potential when accurate operator selection can be maintained even in the presence of hardware noise. However, we may note that one may also bypass the hardware noise during

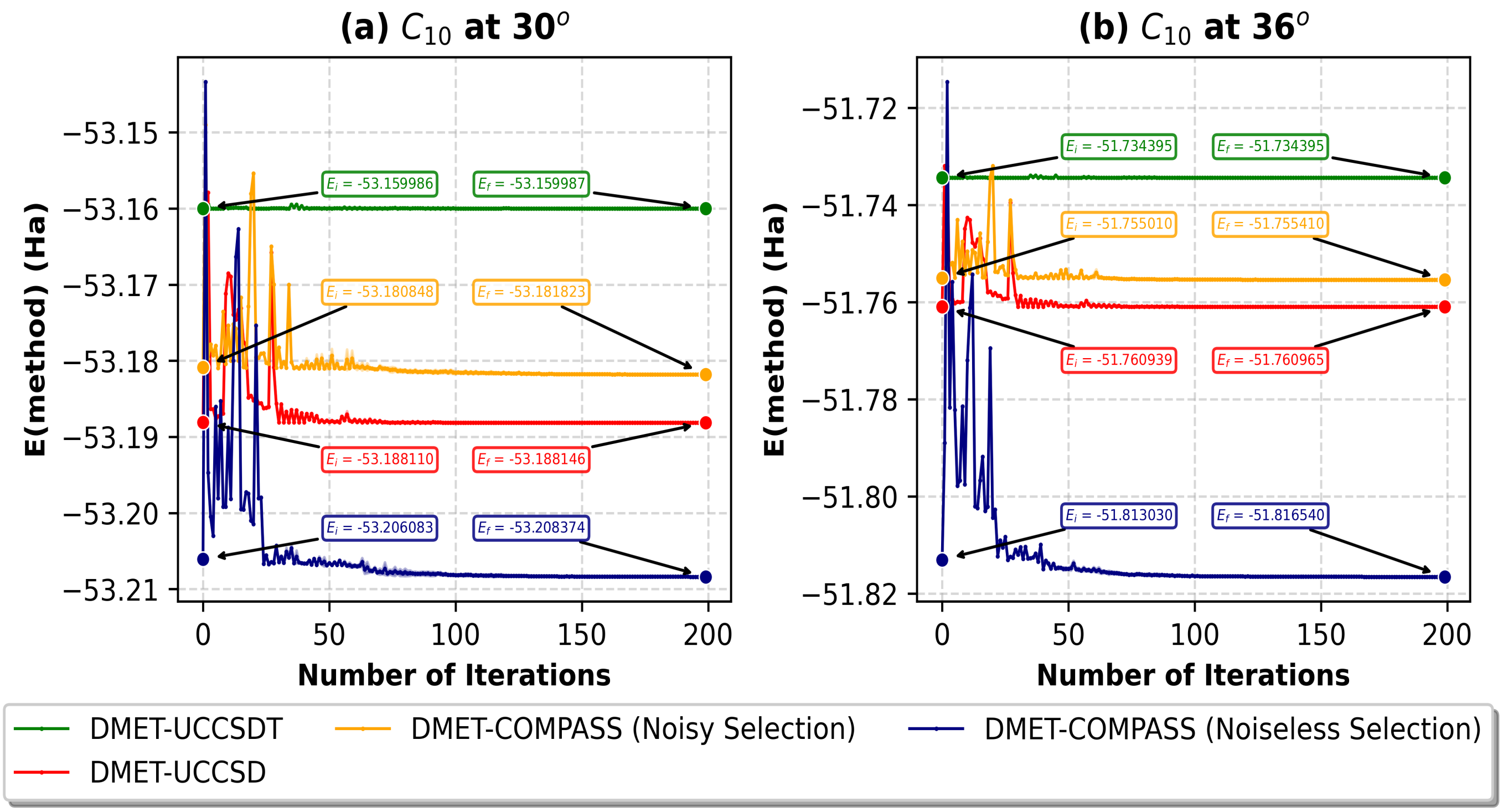

Fig. S5: **The energy convergence of various ansatze during the VQE optimization is shown for two geometries of circular C_{10} in the presence of depolarizing noise. The solid lines represent the mean energy obtained from five independent runs, while the shaded regions (almost indistinguishable from the solid lines) denote the corresponding standard deviations at each optimization step. The values displayed in the boxes indicate the initial and final mean energies achieved during the optimization process.**

the ansatz generation by "classically" constructing a dynamic ansatz that is structurally similar to COMPASS[5], as previously shown by some of us.

[1] Y. Fan, C. Cao, X. Xu, Z. Li, D. Lv, and M.-H. Yung, Circuit-depth reduction of unitary-coupled-cluster ansatz by energy sorting, The Journal of Physical Chemistry Letters **14**, 9596 (2023), pMID: 37862387.

[2] D. Mondal, D. Mukherjee, and R. Maitra, Operator commutativity screening and progressive operator block reordering toward many-body inspired quantum state preparation, The Journal of Chemical Physics **164**, 074113 (2026).

[3] H. R. Grimsley, S. E. Economou, E. Barnes, and N. J. Mayhall, An adaptive variational algorithm for exact molecular simulations on a quantum computer, Nature Communications **10**, 3007 (2019).

[4] S. Halder, A. Dey, C. Shrikhande, and R. Maitra, Machine learning assisted construction of a shallow depth dynamic ansatz for noisy quantum hardware, Chem. Sci. **15**, 3279 (2024).

[5] D. Halder, D. Mondal, and R. Maitra, Noise-independent route toward the genesis of a COMPACT ansatz for molecular energetics: A dynamic approach, The Journal of Chemical Physics **160**, 124104 (2024).
